# How cancer cells remodel lipid metabolism: strategies targeting transcription factors

**DOI:** 10.1186/s12944-021-01593-8

**Published:** 2021-11-14

**Authors:** Do-Won Jeong, Seulbee Lee, Yang-Sook Chun

**Affiliations:** 1 Department of Biomedical Sciences, Seoul National University College of Medicine, Seoul, 03080 South Korea; 2 Department of Physiology, Seoul National University College of Medicine, Seoul, 03080 South Korea; 3 Ischemic/Hypoxic Disease Institute, Seoul National University College of Medicine, Seoul, 03080 South Korea

**Keywords:** Lipid metabolism, Transcription factor, Cancer

## Abstract

Reprogramming of lipid metabolism has received increasing recognition as a hallmark of cancer cells because lipid dysregulation and the alteration of related enzyme profiles are closely correlated with oncogenic signals and malignant phenotypes, such as metastasis and therapeutic resistance. In this review, we describe recent findings that support the importance of lipids, as well as the transcription factors involved in cancer lipid metabolism. With recent advances in transcription factor analysis, including computer-modeling techniques, transcription factors are emerging as central players in cancer biology. Considering the limited number and the crucial role of transcription factors associated with lipid rewiring in cancers, transcription factor targeting is a promising potential strategy for cancer therapy.

**Supplementary Information:**

The online version contains supplementary material available at 10.1186/s12944-021-01593-8.

## Introduction

Cancer is a fundamental disorder of cell proliferation that requires many cellular building blocks, such as proteins, nucleic acids, and lipids. Cancer cells alter metabolism to accumulate metabolic intermediates as sources of these building blocks [1]. The best understood metabolic change is the Warburg effect, which involves exacerbation of glucose uptake and glycolysis [2]. Under normal conditions, glucose undergoes glycolysis to produce pyruvate. The tricarboxylic acid cycle and oxidative phosphorylation then extract energy in the form of adenosine triphosphate. However, in the absence of oxygen, glucose is metabolized to pyruvate, while excess glucose is converted to lactate. Cancer cells tend to prefer the Warburg effect even in the presence of oxygen, leading to increased glucose uptake and consumption, along with decreased oxidative phosphorylation [3]. Another commonly observed metabolic change is glutamine metabolism [4]. Glutamine consumption by cancer cells provides carbon and amino-nitrogen to synthesize nucleotides, amino acids, and lipids [5]. The third hallmark of cancers is altered lipid metabolism. Although changes in lipid metabolism have received less attention than other changes in cancer cells, recent studies have demonstrated a relationship between lipid reprogramming and cancer progression [6].

Lipids are a class of micro-biomolecules that encompass cholesterol (CHO), fatty acids (FAs), and their derivatives (e.g., mono-, di-, and triglycerides [TGs]). Lipids exert multiple biochemical functions in cells, such as membrane synthesis, energy production, and the activation of intracellular signaling pathways. For example, glycerol-lipids are stored lipids used for energy synthesis, while glycerophospholipids are used as structural components of cell membranes. Additionally, sphingolipids serve as signaling molecules to regulate various biological processes, such as cell growth, differentiation, and apoptosis [7]. Because highly proliferating cells require an aberrantly high supply of lipids, the changes in lipid composition and metabolism are considered hallmarks of cancer aggressiveness (e.g., breast, colon, liver and prostate cancers) [8]. Indeed, FA profiles and the levels of TGs and CHO change in the tissues and plasma of patients with various cancers [9–12]. Lipoproteins, such as low-density lipoprotein (LDL), are also higher in the plasma of cancer patients [13]. Importantly, cancer cells obtain the bulk of their lipids from de novo synthesis via transcription factor-dependent regulation, whereas most normal cells acquire their lipids from circulating exogenous lipids [14].

Transcription factors are responsible for the expression of genes needed to adapt to highly proliferating cellular conditions. Recent studies have demonstrated that several diseases are associated with changes in transcription factors. For example, Boyadjiev et al. reported that one-third of human developmental disorders and birth defects can be attributed to dysfunction and gene mutations encoding transcription factors [15]. Additionally, 164 transcription factors have been directly implicated in 277 diseases [16]. In cancers, 294 transcription factors have been identified by comparing a list of 1571 candidate oncogenic proteins with a list of 1988 human transcription factors and regulators [17, 18], which corresponds to approximately 19% of all known oncogenes [19]. In addition, fine-tuning of regulatory systems by transcription factors involved in lipid metabolism helps cancer cells adapt to the challenging microenvironment. For example, upregulation of lipogenic gene expression in cancers results from elevated expression and activation of sterol regulatory element-binding proteins (SREBPs) [20]. Excess carbohydrate intake and hyperglycemia caused by carbohydrate-response element-binding protein (ChREBP) lead to energy storage as TGs and promote tumor progression [21]. Although transcription factors are undruggable targets, an advanced understanding of transcription factors (including their structure) and the dynamics of binding to DNA can provide strategies for fighting cancers.

In this review, we focus on comprehensive insights into lipid metabolism in view of transcription factors, and highlight the complex interplay between lipids and immune system in cancer cells. We propose that lipids and lipid-related transcription factors have the potential to serve as effective therapeutic targets for anticancer immunotherapy.

## Roles of lipids in cancer cells

### Lipids in plasma membranes

Cell membranes contain hundreds of lipids and proteins; they are composed of sphingolipid- CHO-rich membrane rafts known as lipid rafts [22]. Rysman et al. analyzed cellular lipid extracts using a mass spectrometry-based approach and determined the tendency for lipid saturation in clinical tumor tissues, compared with normal tissues. Notably, lipid saturation results from lipid uptake toward de novo lipogenesis, combined with increases in saturated fatty acids (SFAs) and monounsaturated fatty acids (MUFAs), as well as a decrease in polyunsaturated fatty acids (PUFAs). SFAs are less prone to lipid peroxidation, compared with PUFAs [23]. Additionally, SFAs reduce membrane fluidity, resulting in changes in death receptors, apoptotic stimuli, and metastasis in cancer cells [24]. Overall, these changes provide cancer cells with a survival benefit.

### Lipids as signaling molecules

Lipids act as intra- and extracellular messengers; they can become potent mediators of malignant behavior. Sphingolipids are a class of lipids that contain ceramide and sphingosine, and they have multiple roles in cancer cell survival [7]. For example, sphingosine-1-phosphate, which is produced from sphingosine, increases cell proliferation and tumor malignancy by activating both signal transducer and activator of transcription 3 (STAT3) and the Warburg effect [25, 26]. Phosphoinositides are central mediators of the phosphoinositide 3-kinase (PI3K)/Akt/mammalian target of rapamycin (mTOR) signaling axis. PI3K activates the rapid conversion of phosphatidylinositol (PI; 4,5) P2 into PI (3,4,5) P3, which leads to the recruitment and activation of Akt [27]. PIP3 is also the substrate for phosphatase and tensin homolog (PTEN), and PTEN is most frequently mutated or deleted in cancer [28]. Increased levels of lysophosphatidic acid receptors have been described in several cancers; these increased levels contribute to cell invasiveness [29]. Lysophosphatidic acid is produced by the lysophospholipase autotaxin; it activates cell proliferation and tumor invasiveness by binding to G-protein-coupled receptors [30]. Prostaglandin E2 is an eicosanoid [31] that activates the Ras pathway and induces cell proliferation, which is associated with a poor prognosis [32]. Therefore, lipids are involved in multiple cellular signaling processes, many of which are linked to oncogenesis.

### Lipids as protein modulators

**Fig. 1:**
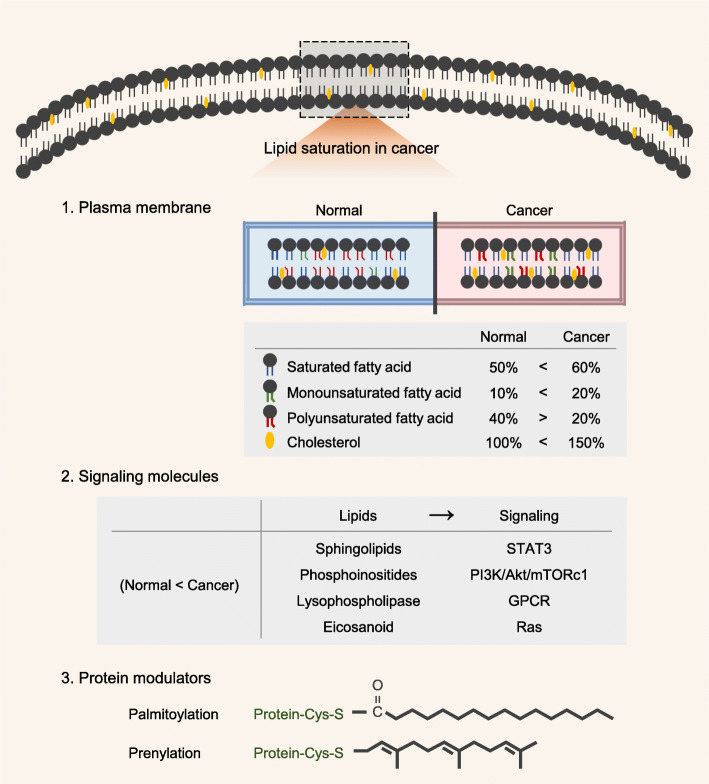
Diagrammatic sketch of the role of lipids in cancers

Lipids regulate proteins by dynamic lipid post-translational modifications. Among these modifications, palmitoylation has attracted considerable interest, because it is essential for the functioning of key signaling oncoproteins [33]. For example, palmitoylation is required for Wnt secretion because it facilitates the interaction between Wnt and its intracellular chaperone Wntless [34]. The key oncoprotein Ras is also palmitoylated, thus facilitating membrane localization of Ras [35]. Epidermal growth factor receptor palmitoylation promotes PI3K/Akt signaling, leading to cell proliferation in lung cancer [36]. Another type of lipid-related post-translational modification is prenylation, in which the farnesyl group is covalently attached to target proteins [37]. Drugs targeting prenyltransferases have been used in preclinical trials; they show protective effects in several solid cancers [38]. Guanosine triphosphate (GTP) enzymes (e.g., Cell division cycle 42, G-protein-coupled receptors, Ras, and Pho) may be prenylated, which is crucial for their membrane association and activation [39]. Indeed, the deletion of prenyltransferases inhibits the association between the Ras family and the plasma membrane; this delays Ras-induced lung tumor formation [40]. The role of lipids in cancers is illustrated in Fig. 1.

## Lipid reprogramming in cancer

### Changes in the lipid profile and cancer development

Recent advances in technologies including tandem mass spectrometry, RAMAN scattering microscopy, and electrospray ionization have enabled the quantitative analysis of lipids [41]. Differences in lipid profiles between malignant tumor specimens and matched normal tissues indicate that lipids are potential diagnostic biomarkers. For example, Qiu et al. investigated phospholipid composition in plasma samples of patients with hepatocellular carcinoma (HCC); they detected increases in SFAs and MUFAs. However, they found that PUFAs decrease in the plasma of HCC patients [42]. A decrease in linoleic acid and an increase in OA were found in patients with bladder cancer [43], lymphoma [44], and colorectal cancer [9]. An abundance of diverse lysophospholipids is present in colorectal cancer, compared with benign counterpart tissue [10]. Another study revealed reduced levels of TGs, stearic acid, myristic acid, palmitic acid, and OA in colorectal cancer patients; total phospholipids and PUFAs were increased in those patients [11]. Children with lymphoblastic leukemia have higher serum levels of TGs and LDL [13]. Similarly, plasma CHO and LDL are higher in breast cancer patients than in controls. Thysell et al. demonstrated higher levels of CHO in prostate cancer patients with bone metastasis than in other types of cancer patients with bone metastasis from different tissues, as well as normal bone [12]. Changes of the lipid profile in cancers are presented in Table 1.

**Table 1:** Diagnostic lipid signatures in cancers

Cancer type	Control samples	Cancer samples	Biofluid	Altered lipids	References
Hepatocellular carcinoma	14	23	Plasma	SFA (12:0, 14:0, 15:0, 16:0, 20:0, 22:0) MUFA (16:1n-7, 17:1n-9, 18:1n-9, 22:1n-9) PUFA (18:2n-6, 20:4n-6, 22:5n-3)	[42]
	42	42	Tissue	SFA (14:0, 15:0) MUFA (14:1, 16:1n-7, 17:1n-9, 18:1n-9, 24:1n-9) PUFA (18:2n-6, 20:3n-6, 22:2n-6, 22:4n-6, 22:5n-3, 22:6n-3)	
Bladder cancer	31	31	Tissue	SFA (4:0, 6:0, 7:0, 8:0, 16:0) MUFA (16:1n-7, 20:1n-9) PUFA (18:2n-6, 18:3n-3, 18:4n-3 20:1n-9, 20:2n-6, 20:5n-3, 22:2n-6, 22:3n-3, 22:5n-3, 22:6n-3), CHO	[43]
Lymphoma	29	47	Plasma	SFA (16:0) MUFA (18:1n-9) PUFA (18:2n-6, 20:5n-3, 22:4n-6, 22:6n-3)	[44]
Colorectal cancer	12	17	Plasma	SFA (16:0) MUFA (18:1n-7, 18:1n-9) PUFA (18:2n-6, 20:3n-6) TG, CHO	[9]
	20	20	Tissue	SFA (14:0, 18:0) MUFA (16:1, 18:1) PUFA (18:2, 20:4, 20:5, 22:2, 22:3) TG, CHO	[10]
	25	25	Tissue	SFA (16:0, 18:0) MUFA (16:1, 18:1) PUFA (18:3, 20:4, 20:5, 22:6) TG, CHO	[11]
Lymphoblastic leukemia	22	80	Plasma	TG, LDL	[13]
Prostate cancer	14	20	Plasma	SFA (18:0) PUFA (18:2n-6, 20:4) TG, CHO	[12]

*SFA* saturated fatty acids; *MUFA* monounsaturated fatty acids; *PUFA* polyunsaturated fatty acids; *TG* triglyceride; *CHO* cholesterol; *LDL* low-density lipoprotein; *HDL* high-density lipoprotein

Polar lipids, multiple phospholipids, and other metabolites are also upregulated in prostate cancer cell lines [45]. Other studies have shown that high levels of SFAs and MUFAs are present in association with high metastatic potential in breast and melanoma cell lineages [46, 47]. Diglyceride is overexpressed in metastatic osteosarcoma cells; diglyceride targeting reduces cell migration, providing a clue that pharmacological targeting of certain lipids could be therapeutic [48]. Changes in the lipid profiles of various cancers are summarized in Table 1.

### Abnormal levels of lipogenic enzymes in cancer

More than 90% of lipids required by cancer cells are reportedly derived from de novo synthesis. Consistent with these published findings, cancer cells often show upregulation of de novo lipogenic enzymes involved in the synthesis of FAs and CHO. For example, adenosine triphosphate citrate lyase and acetyl-coenzyme A carboxylase are upregulated and activated in most cancers [49]. Fatty acid synthase (FASN) is a metabolic oncogene, because elevated levels of FASN are detected in various solid tumors, including breast, prostate, colon, lung, bladder, ovary, stomach, endometrium, kidney, skin, esophagus, and tongue [50]. In addition, the upregulation of FASN is associated with unfavorable outcomes in patients with the most aggressive tumors. Abnormally elevated CHO levels in cancer cells result from 3-hydroxy-3-methylglutaryl coenzyme A reductase. High expression of 3-hydroxy-3-methylglutaryl coenzyme A reductase has been associated with poor pathological features and survival in breast cancer patients [51]. Overall, aberrantly high levels of lipids and lipogenic enzymes are significantly associated with the development, progression, and poor prognosis of several cancers.

## Transcription factors leading to lipid metabolism rewiring in cancer

### ChREBP

Glucose and other simple sugars obtained from the diet are metabolized to provide acetyl-CoA for the synthesis of FAs. During this process, ChREBP, encoded by the *MLXIPL* gene, acts as a transcriptional mediator to convert excess glucose to fat in the liver [52]. ChREBP binds to the carbohydrate-response elements of glycolytic and lipogenic genes, such as liver-type pyruvate kinase, *FASN*, and acetyl-coenzyme A carboxylase; through these interactions, ChREBP coordinates the carbohydrate induction of lipogenesis. Increased hepatic glucose accumulation and decreased hepatic FAs are detected in ChREBP knockout mice, compared with wild-type mice [53].

ChREBP has major roles in the pathogenesis of different types of cancer. ChREBP knockdown by siRNA significantly inhibits aerobic glycolysis and the synthesis of lipids in colon cancer cells. Additionally, ChREBP knockdown activates p53, induces cell cycle arrest, and reduces colon cancer growth in vivo, indicating an oncogenic function for ChREBP [54, 55]. Positive correlations of *MLXIPL* mRNA with glycolytic and lipogenic genes were also observed in a comprehensive chromatin immunoprecipitation analysis of human HCC and breast cancer. Glucose transporter 1 increases significantly in HCC patients, and its expression is positively correlated with ChREBP. In contrast, the opposite association has been observed between the ChREBP protein and pyruvate dehydrogenase kinase 2 genes, which inactivate acetyl-CoA production [56]. Cancer cells favor the conversion of pyruvate into acetyl-CoA, rather than the accumulation of pyruvate [57]. Considering that the ChREBP protein is positively correlated with tumor malignancy [56], these results show that the oncogenic role of ChREBP/*MLXIPL* results from the conversion of glucose to fat [58].

### FXR

Farnesoid X receptor (FXR), encoded by the *NR1H4* gene, was originally identified as a nuclear receptor activated by farnesol metabolites [59]. However, more recent studies have revealed that FXR is an endogenous bile acid-receptor that contributes to the maintenance of CHO/bile acid homeostasis by regulating various metabolic enzymes [60]. Because bile acids constitute a major factor that facilitates the absorption of dietary fats and steroids, the functions of FXR in metabolic diseases are well-established [61]. For example, FXR-null mice develop elevated serum CHO and TGs levels; however, they also show reduced adipocyte size and protective effects against high-fat diet (HFD)-induced obesity [62]. Genetic depletion of intestinal FXR in mice markedly decreases HFD-induced insulin resistance and fatty liver because of reductions in ceramide levels in the intestines and serum [63]. Although FXR-deficient mice are resistant to HFD-induced obesity, FXR agonists protect the liver from inflammation and fibrosis in the non-alcoholic steatohepatitis mouse model [64].

Similarly, FXR has crucial roles in the pathogenesis of several cancers. Loss of FXR function promotes intestinal fat absorption and synergistically increases liver carcinogenesis [65]. In mice, HFD-induced bile acid accumulation inhibits FXR function and subsequently leads to the progression of colorectal cancer. In contrast, the activation of intestinal FXR by a selective agonist restricts colon cell growth, regardless of HFD conditions [66]. Lipids isolated from bone induce the migration of breast cancer cells; FXR modulates this tumorigenic effect of bone-derived lipids, and eventually regulates the metastasis of breast tumor cells to bone [67]. Anakk et al. reported that loss of the FXR disrupts bile acid metabolism; it leads to activation of the Yes-associated protein and subsequent hepatocarcinogenesis [68]. Because studies of the FXR-mediated metabolic pathways have mainly focused on bile acid signaling, the mechanisms regulated by FXR in cancers require further investigation.

### LXRs

Liver X receptors (LXRs) have been an intriguing target for the treatment of inflammation, Alzheimer’s disease, and cancer. LXRs are related to nuclear receptors (e.g., PPAR, FXR, and RXR) and are classified as a subfamily of *NR1H3* (LXRα) and *NR1H2* (LXRβ) [69]. LXRα is mainly expressed in liver tissue [70], whereas LXRβ is expressed in most tissues [71]. LXRs are upstream of the SREBP1c and ChREBP proteins; they directly regulate glycolysis and increase lipogenesis [72]. Compared with wild-type mice, LXR knockout mice show decreased levels of key lipogenic genes, including *SREBP1c*, *FASN*, and stearoyl-CoA desaturase1 (*SCD1*), as well as decreased production of TGs [73].

LXRs are CHO sensors that exert anticancer effects in various cancers. For example, LXR-driven genes, such as adenosine triphosphate binding cassette subfamily G member 1 (*ABCG1*) and apolipoprotein E, are expressed less frequently in breast cancer. The *ABCG1* and apolipoprotein E proteins are involved in CHO efflux from the cell to extracellular acceptors; the loss of LXR-mediated genes results in higher lipid contents in cells, as well as greater cancer cell viability. Similarly, LXR agonists degrade the LDL receptor and increase the expression of the adenosine triphosphate binding cassette subfamily A member 1 (ABCA1) CHO efflux transporter, preventing exogenous CHO uptake in glioblastoma. Because glioblastoma cells require high levels of CHO for growth, LXR agonists promote tumor cell death [74]. Another LXR agonist induces increases in the *ABCG1* level and subsequently stimulates reverse CHO transport in prostate cancer cells. Atomic force microscopy scanning of the plasma membrane has revealed thinner lipid rafts after LXR stimulation. Thus, LXR agonists suppress the growth of prostate cancer cells in both xenografted nude mice and cell culture [75]. Furthermore, activation of LXRs decreases the expression of lipogenic genes (e.g., *SREBP1c*, *SCD1*, and *FASN*) in breast cancer cells. LXR agonists subsequently suppress cell cycle genes, indicating an anti-proliferative role for LXRs [76]. However, LXR antagonists inhibit both the Warburg effect and lipogenesis, thereby inducing apoptosis in colon, lung, and prostate cancer cells; notably, these antagonists have no toxic effects on non-malignant cells [77]. Considering that LXR agonists and antagonists show different effects, the development of a strategy for LXRs (*NR1H3* and *NR1H2*) as an anticancer drug should be carefully considered.

### PPARs

Peroxisome proliferator-activated receptors (PPARs) are transcription factors that control gene expression by binding to peroxisome proliferator response elements in the promoters of target genes [78, 79]. According to their tissue distribution and ligand specificity, PPARs are divided into three nuclear receptor subtypes: -α, −β/δ, and -γ [80]. PPARα is mainly expressed in tissues with high rates of FA β-oxidation, such as the liver, kidneys, heart, and muscle. PPARβ/δ is ubiquitously expressed in most tissues, and PPARγ is mainly expressed in adipose tissue [79, 81]. These PPAR isoforms modulate lipid metabolism in different manners. PPARα is a main energy-producing factor during nutrient deficiency and participates in FA β-oxidation. Additionally, PPARα downregulates hepatic apolipoprotein A-I and C-III; it also increases lipoprotein lipase gene expression, leading to a decrease in plasma TGs [82]. PPARβ/δ induces glucose 6-phosphate dehydrogenase activity, increases FA β-oxidation in muscle, and inhibits the release of FAs from white adipose tissue [83]. In diet-induced obese mice, the activation of PPARβ/δ normalizes serum insulin and TGs concentrations; it also acts as a new target for the treatment of type 2 diabetes [84]. Similarly, PPARβ/δ protects pancreatic islets against FA-induced β-cell dysfunction [85]. PPARγ promotes energy storage by directing FAs toward esterification and accumulation as TGs [86]. Dietary supplementation of the PPARγ agonist rosiglitazone suppresses type 2 diabetes in obese mice, but chronic treatment with rosiglitazone markedly aggravates hepatic steatosis [87].

In addition, clinically or preclinically relevant relationships between PPARs and cancers have been observed. Pan-cancer datasets of patients with 21 cancers show that altered PPARs signaling dysregulates numerous tumor cell lipid metabolic-related pathways to directly impact patient survival [88]. Moreover, mice fed a PPARα agonist exhibit lower body weights and an increased incidence of hepatic carcinogenesis. Tumors and visible nodules up to approximately 11 mm in diameter were apparent in livers from PPARα agonist-fed mice [89]. PPARδ induces xenograft tumor growth of prostate cancer cells by regulating the ATP binding cassette transporter 1 (*ABCA1*) gene. *ABCA1* is a CHO efflux-related gene; PPARδ directly increases the levels of *ABCA1* mRNA and membrane CHO expression, which is followed by tumor growth [90]. PPARγ has tumor-suppressive and oncogenic effects in several cancers. In lung cancer cells, PPARγ-mediated lipid synthesis strongly induces mitochondrial reactive oxygen species stress and contributes to tumor suppression [91]. In addition, the chemical activation of PPARγ increases fatty acid binding protein 4 (FABP4) expression, which is accompanied by increased levels of intracellular reactive oxygen species in lung cancer cells. PPARγ-driven induction of FABP4 and lipoprotein lipase results in a better prognosis for lung and renal cancer patients [92]. In contrast, the interaction between PPARγ and Nur77 plays an antagonistic role in breast cancer. Nur77 recruits PPARγ to the CD36 promoter and FABP4 to suppress transcription of these genes, thus preventing FA uptake and cell proliferation. PPARγ physically binds to Nur77 and facilitates ubiquitin ligase Trim13-mediated ubiquitination of Nur77, thereby aggravating breast cancer [93].

Notably, dietary FAs directly bind to PPARs and mimic the effects of synthetic agonists that activate PPARs [94]. In this manner, PPARs sense the FA signals derived from dietary lipids and serve as lipid modulators to facilitate cancer progression. For example, an HFD promotes tumor cell growth and metastasis of colorectal cancer cells to the liver in mice. However, PPARδ deletion in mice completely inhibits the effect of an HFD, along with expression of Nanog and CD44, demonstrating that PPARs mediate the tumorigenic effect of HFD on cancer progression [95].

### SREBPs

Lipid homeostasis is regulated by SREBP transcription factors. SREBPs directly activate the expression of more than 30 genes; they also contribute to the synthesis and uptake of CHO, FAs, and TGs. The N-terminal domain of SREBP must be proteolytically processed to act as a transcription factor. When cells experience CHO depletion, the SREBP cleavage activation protein (SCAP) escorts the SREBP from the endoplasmic reticulum to the Golgi apparatus. The SREBP is subsequently cleaved in the Golgi apparatus [96], and the mature SREBP translocates to the nucleus where it binds to a sterol response element in the promoter of the target gene [97]. The mammalian genome encodes three SREBP isoforms: SREBP1a, SREBP1c, and SREBP2 [98]. The same gene on human chromosome 17p11.2 encodes SREBP1a and SREBP1c. However, SREBP2 is encoded by distinct genes on human chromosome 22q13 [99]. SREBP1a is a potent activator of all SREBP-responsive genes, including genes involved in the synthesis of LDL receptor, CHO, and FAs; SREBP1c preferentially enhances the expression of genes related to FA synthesis. Additionally, cells produce both SREBP1a and − 2 to activate CHO synthesis in the presence of increased demands for CHO [97].

Recent studies have shown that SREBPs regulate lipid metabolism in cancers. Inhibition of SREBPs at the transcriptional level attenuates the expression of lipogenic genes and lipid uptake in patients with ovarian cancer. In this context, silencing of the SREBP genes abrogates ovarian tumor growth, blood vessel formation, and lipid content both in vitro and in vivo [100]. Consistent with these published findings, silencing of SREBP downstream genes showed that de novo FA synthesis and membrane phospholipids are required for breast and pancreatic cancer growth [24, 101]. SREBPs enhance the prenylation of N-Ras, leading to its activation and oncogenic effects [102].

Studies targeting the SREBP maturation pathways in cancers are well-established. Li et al. reported that pharmacological inhibition of SREBP proteolysis reduces HCC progression by regulating FA and CHO metabolism [103]. In various cancer cell lines, such as HeLa, T98, and U2OS, inhibition of SREBP-SCAP complex transport from the endoplasmic reticulum to the Golgi apparatus reveals anti-tumor properties. In addition to suppressing lipid metabolism, the inhibition of SREBP maturation perturbs tubulin polymerization and mitotic spindle assembly, leading to decreased cancer cell proliferation and migration [104].

**Fig. 2:**
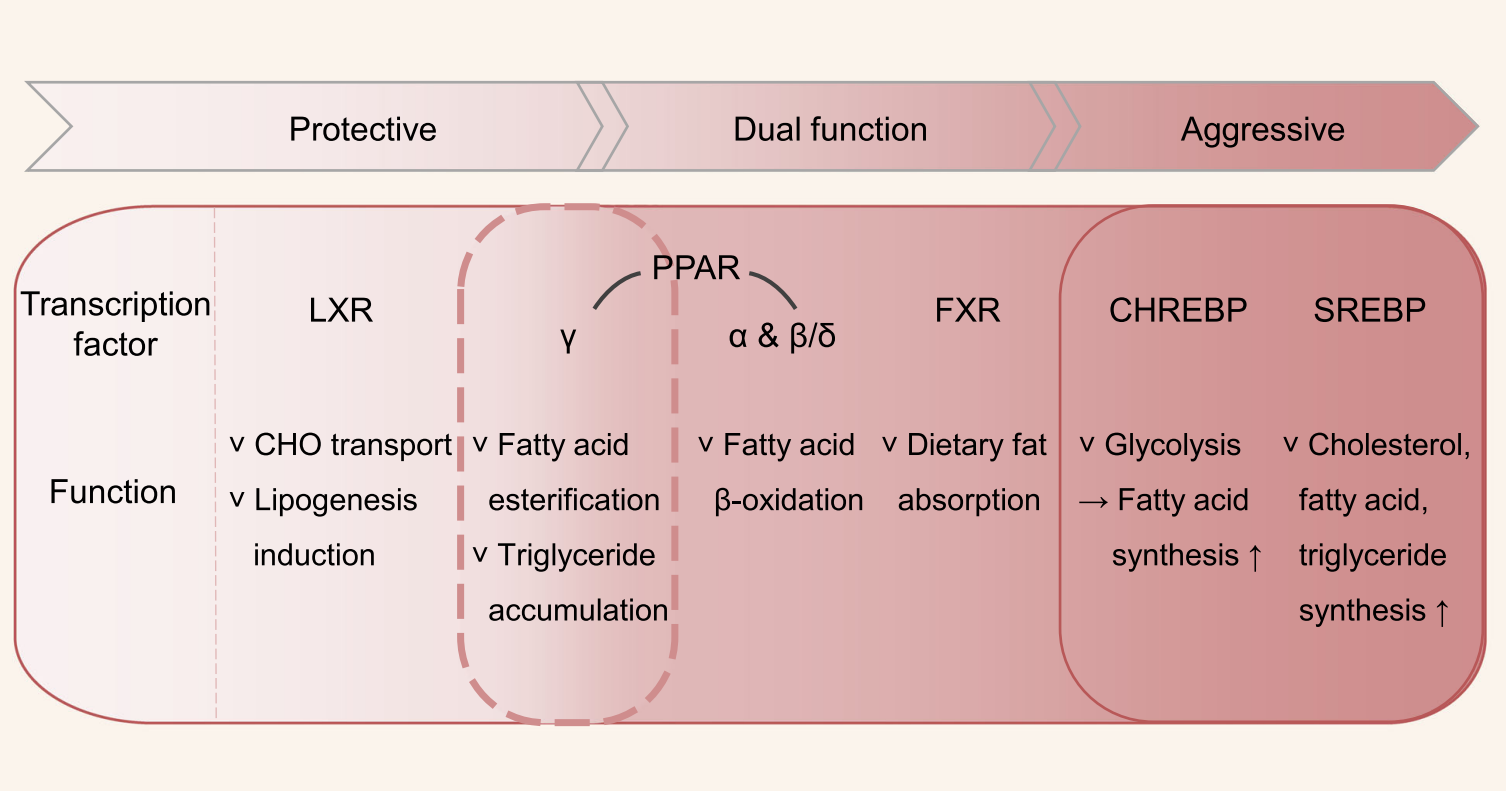
A summary of the role of lipid-related transcription factors in cancer cells

SREBPs also provide survival advantages to cancer cells. Nuclear SREBP1 is correlated with high LDL receptor levels in glioblastoma patients. Because cancer cells use high levels of CHO for growth, SREBP-driven upregulation of the LDL receptor prevents apoptotic cell death [74]. In addition, SREBPs protect cancer cells from reactive oxygen species and endoplasmic reticulum stress by altering the ratio of SFAs to unsaturated long-chain FAs [105]. Because SFAs are less lipotoxic than PUFAs, SREBPs allow cancer cells to survive the harsh microenvironment [23]. These findings emphasize that SREBPs and their regulatory systems could serve as potential therapeutic targets. The role of lipid-related transcription factors in cancer is presented in Fig. 2.

## Therapeutic strategies targeting lipid metabolism

Due to the central role of lipids and related transcription factors in cancer, continuous efforts have been made to adjust lipid metabolism as anticancer drugs. This approach was employed in preclinical models both in vitro and in vivo, and some of the drugs have entered clinical trials. Interestingly, lipids themselves showed promising effects on cancer. Here, we listed a brief overview of therapeutic drugs targeting transcription factors and lipid-related enzymes and lipids.

### Drugs targeting FXR

GW4064 is a synthetic FXR agonist that increases cell proliferation and invasion by activating NF-κB and N-cadherin in HCC and pancreatic cancer cells [106, 107]. However, some drugs that target FXR have shown a protective effect on tumors. GW4064 inhibits proliferation and migration and induces apoptosis in esophageal and liver cancer [108, 109]. The FXR antagonist, guggulsterone, inhibits migration in liver cancer and pancreatic cancer by regulating NF-kB [106, 107]. Since the effectiveness of drugs targeting FXR varies with the type of cancer tissue, treatments should be designed for specific organs.

### Drugs targeting LXR

The LXR inhibitor, SR9243, inhibits the Warburg effect and lipogenesis, and induces apoptosis of cancer cells, but is not toxic to non-malignant cells [77]. Pommier et al. The elucidated LXR activator T0901317 down-regulates the Akt pathway and induces apoptosis in vivo and in prostate cancer cells [75]. The LXR agonist GW3965 induces cell death by degrading LDL receptors and increasing the expression of ABCA1 CHO efflux transporter [74]. Another LXR agonist, LXR-623, crosses the blood-brain barrier and reaches therapeutic levels in glioblastoma cells [110].

### Drugs targeting PPARs

The PPARα agonist, fenofibrate, inhibits tumor growth and angiogenesis in melanoma and fibrosarcoma [111]. In breast cancer, fenofibrate promotes chemotherapy sensitivity by down-regulating Mcl-1 and Bcl-xl and up-regulating Bok and Bax at the transcriptional level [112]. Fenofibrate reduces the migration of oral cancer cells and promotes the autophagy of prostate tumors in vivo by interfering with the Warburg effect and regulating the adenosine monophosphate-activated protein kinase (AMPK)-mTOR pathway [113, 114]. In addition, as a selective PPARα ligand, Wy-14,643 down-regulates cytochrome P450 CYP2C, an enzyme that catalyzes the epoxidation of PUFA, and also inhibits endothelial cell proliferation and tumorigenesis [115, 116]. However, chronic treatment of Wy-14,643 increases the incidence of liver cancer through induction of oxidative stress in vivo [89].

Genetic suppression of PPARδ inhibits tumor growth of prostate cancer cells [90], however, some of the studies have reported the anticancer activity of PPARβ/δ agonists. Zaveri et al. suggested that PPARβ/δ antagonist, SR13904, inhibits the proliferation of the lung cancer cell [117]. PPARβ/δ agonist, GW0742, can improve the efficacy of preventing skin tumors, especially when combined with cyclooxygenase 2 inhibitors [118, 119].

Thiazolidinedione (also known as glitazones) is newly developed synthetic ligand of PPARγ. Mechanistically, thiazolidinedione directly binds and leads to conformational change in PPARγ, and activates the transcriptional machinery. PPARγ agonists are used clinically as antidiabetic agents, resulting in sensitizing insulin and lowering blood glucose concentration [120]. Besides the metabolic actions, thiazolidinedione leads to cell death by targeting cyclin-dependent kinase in malignant cell lines (e.g., colon, liver and pancreatic cancer) [121–125]. Thiazolidinedione decreases the release of endothelial growth factor from smooth muscle cells and inhibits angiogenesis in glioblastoma, liposarcoma, lung and prostate cancer [126, 127]. Importantly, epithelial-mesenchymal transition-derived breast cancer cells can differentiate into post-mitotic adipocytes, and lose their invasive and oncogenic phenotypes. Thiazolidinedione forces trans-differentiation of cancer cells into adipocyte and prevents tumor invasion and metastasis. PPARγ antagonists, T0070907 and GW9662, have protective effects on bladder cancer cells [128]. However, drugs targeting PPARs have not been conclusive on the beneficial effect for patients [129, 130]. Clinical trials need more research.

### Drugs targeting SREBPs

Silibinin is isolated from the seeds of the milk thistle plants, and mechanistically increases SREBP1 phosphorylation and inhibits its nuclear translocation. In breast cancer cells, silibinin promotes autophagy by inhibiting estrogen receptor-α and upregulating estrogen receptor-β [131]. Silibinin inhibits cell invasion by decreasing the production of urokinase-plasminogen activator and matrix metalloproteinase-2 in human lung cancer cells [132]. Silibinin also showed strong effect on head and neck and prostate cancer [133–135].

Betulin, fatostatin and nelfinavir suppress the processing of SREBPs and have anti-tumor properties. Li et al. found that betulin dramatically reduces diethylnitrosamine-induced HCC progression by downregulating tumor promoting cytokines such as interleukin-6 and -1β [103]. Fatostatin inhibits cell proliferation and invasion both in vitro and in vivo (e.g., glioblastoma, osteosarcoma, breast and prostate cancer) [104, 136, 137]. Compound 24 is a derivative of fatostatin, which was developed to improve chemical properties in a variety of disease models [138]. Compound 24 decreases the levels of FAs and cellular growth in glioma, prostate, and breast cancer cell lines [105]. Nelvinavir was approved by the Food and Drug Administration for HIV treatment in humans at first, and is currently in phase II clinical trials for myeloma, glioblastoma, pancreatic and lung cancer. In vitro, nelfinavir inhibits cell proliferation in liposarcoma and prostate cancer cells [139, 140].

### Drugs targeting lipid metabolism

FASN inhibitors have been the subject of many studies and one of the drugs that have entered clinical trials is TVB-2640. TVB-2640 showed significant anti-cancer activities across multiple tumor types including lung, ovarian, and breast cancer [141]. C75, another compound targeting FASN, directly increases FA oxidation and results in apoptosis during S phase in breast cancer cells [142]. Cerulenin reduces the transactivation of estrogen receptors in ovarian cancer, and suppresses liver metastasis of colon cancer, and induces cancer cell death both in vivo and in vitro [143, 144]. Orlistat is a drug approved for obesity management by the Food and Drug Administration in 1999. Orlistat induces endoplasmic reticulum stress and subsequently increases apoptosis in breast, colon and prostate cancer cells [145, 146].

Preclinical investigations are ongoing on the use of an inhibitor of acetyl-CoA carboxylase (ACC). Soraphen A induces cell growth arrest and cytotoxicity in prostate cancer cells [147]. ND-646 is an allosteric inhibitor of ACC, which inhibits the growth of non-small cell lung tumor growth in vitro and in vivo [148]. Another allosteric inhibitor of ACC, TOFA, induces cell death by releasing proapoptotic proteins from mitochondria in breast, colon, lung and ovarian cancer [149–151]. SCD1 inhibitor A939572 induces endoplasmic reticulum stress and enhances cell death in clear cell renal cell carcinoma [152]. A939572 decreases the phosphorylation of the PI3K/Akt pathway and significantly suppresses the cell vitality of lung cancer cells in vivo [153]. Blockage of SCD1 with CAY10566 induces cell apoptosis by inactivating the AMPK signaling in HCC [154]. CAY10566 also showed protective effect on glioblastoma, melanoma, ovarian and lung cancer [155–157].

Targeting CHO synthesis could be another potential approach. Statin is a β-hydroxy β-methylglutaryl-CoA reductase (HMGCR) iinhibitor and has shown promising outcomes in vitro and in vivo. Statin inhibits lipid metabolism and leads to cell survival in various cancers (e.g., colon, pancreatic, liver, breast, prostate, bladder, lung and skin cancer) [158, 159]. In 3129 human epidemiologic studies, the use of statin reduces incidence and recurrence of various cancers (e.g., bladder, breast, colon, kidney, lung, skin, pancreas and prostate cancer) [160–163]. Another CHO synthesis inhibitor simvastatin also inhibits the Akt pathway and induces apoptosis in prostate cancer cells [164]. However, a recent meta-analysis of cancers revealed no significant effect of statin on cancer therapy indicating that the clinical use of drugs targeting CHO should be carefully considered [165, 166].

The challenge of targeting lipid uptake is focused on CD36 and FABPs. ABT-510 is a synthetic analog of thrombospondin-1 and reaches phase I clinical studies against glioblastoma, melanoma and renal cell carcinoma [167–169]. Mechanistically, ABT-510 directly binds to CD36 and induces the death receptor Fas expression [170, 171]. In addition, Al-Jameel et al. produced a highly efficient recombinant FABP5 inhibitor, named dmrFABP5. FABP5 binds and transports medium and long chain FAs into the nucleus of cancer cells. dmrFABP5 is synthesized by switching three amino acids of FABP5, losing its ability to bind FAs. dmrFABP5 significantly suppresses proliferation, migration, and invasion of prostate cancer cells [172]. SBFI-102 and SBFI-103 increase cytotoxicity in prostate cancer cells and stimulate tumor-suppressive effects of other chemicals, taxanes [173]. EI-05, FABP5 activator, enhances lipid droplet and interferon-β production, which further promotes the anti-tumor activity of macrophages during inflammation. In breast cancer cells, administration of EI-05 in vivo significantly inhibits cell growth [174]. The list of lipid-related drugs is summarized in Table 2.

**Table 2:** List of lipid-related drugs

Target	Drug	Phase of development	Cancer type	Reference
FXR	GW4064	Preclinical	Eesophagus, liver and pancreatic cancer	[106–109]
	Guggulsterone	Preclinical	Liver and pancreatic cancer	[106, 107]
LXR	GW3965	Preclinical	Glioblastoma	[74]
	LXR-623	Phase I	Glioblastoma	[110]
	SR9243	Preclinical	Colon cancer	[77]
	T0901317	Preclinical	Prostate cancer	[75]
PPARα	Fenofibrate	Preclinical	Breast, oral and prostate cancer, Melanoma	[111–114]
	Wy-14,643	Preclinical	Breast, colon, lung and liver cancer	[89, 115]
PPARβ/δ	SR13904	Preclinical	Lung cancer	[117]
	GW0742	Preclinical	Melanoma	[118, 119]
PPARγ	Thiazolidinedione	Phase III	Colon, pancreatic, liver and breast cancer, Liposarcoma	[121–126]
	GW9662	Clinical	Bladder cancer	[128]
SREBPs	Sibilinin	Preclinical	Breast, head and neck, lung, and prostate cancer	[131–135]
	Betulin	Preclinical	HCC	[103]
	Fatostatin	Preclinical	Glioblastoma, osteosarcoma, breast and prostate cancer	[104, 136, 137]
	Compound 24	Preclinical	Glioblastoma	[105]
S1P/S2P	Nelfinavir	Phase II	Glioblastoma, liposarcoma, myeloma, lung, pancreatic, and prostate cancer	[139, 140, 175]
FAS	TVB-2640	Phase II	Lung, ovarian and breast cancer	[141]
	C75	Preclinical	Breast, colon, ovarian, and prostate cancer	[142, 144, 145, 176]
	Cerulenin	Preclinical	Colon and ovarian cancer	[143, 144]
	Orlistat	Approved for anti-obesity	Breast, colon and prostate cancer	[145, 146]
ACC	Soraphen A	Preclinical	Prostate cancer	[147]
	ND-646	Preclinical	Lung cancer	[148]
	TOFA	Preclinical	Breast, colon, lung, and prostate cancer	[149–151]
SCD	A939572	Preclinical	Clear cell renal cell carcinoma, Lung cancer	[152, 153]
	CAY10566	Preclinical	Colon and ovarian cancer, glioblastoma, melanoma, HCC	[154–157]
HMGCR	Statin	Approved	Many cancers	[158–163, 165, 166]
	Simvastatin	Preclinical	Prostate cancer	[164]
CD36	ABT-510	Phase I	Glioblastoma, melanoma and renal cell carcinoma	[167–169]
FABP5	dmrFABP5	Preclinical	Prostate cancer	[172]
	SBFI-102 and SBFI-103	Preclinical	Prostate cancer	[173]
	EI-05	Preclinical	Breast cancer	[174]

*FXR* Farnesoid X receptor; *SREBP* Sterol regulatory element-binding protein; *S1P* site-1 protease; *LXR* Liver X receptor; *ChREBP* Carbohydrate-response element-binding protein; *PPARs* Peroxisome proliferator-activated receptor; *FASN* Fatty acid synthase; *ACC* Acetyl-coenzyme A carboxylase; *SCD* Stearoyl-CoA desaturase; *HCC* Hepatocellular carcinoma; *HMGCR* β-Hydroxy β-methylglutaryl-CoA reductase

## Dietary lipids and cancer

Because excess calorie intake and obesity are linked to a high risk of cancer aggressiveness, many studies have focused on dietary adjustments as a potential target for cancer therapy [177]. An HFD has been regarded as a common pathogenic factor in many diseases. However, recent studies have identified that the effect of dietary fat depends on the composition of individual FAs. SFAs are positively associated with carcinogenesis, whereas n-3 PUFAs are more likely to have protective effects against cancer [178, 179]. Data regarding the effects of MUFAs and n-6 PUFA on cancer progression are controversial.

The protective effects of n-3 PUFAs have been confirmed in numerous cancer cell lines. Docosahexaenoic acid is a major n-3 PUFA involved in anticancer activity. In vitro studies have demonstrated that docosahexaenoic acid inhibits cancer cell proliferation and resistance to irradiation by regulating the Akt and Wnt pathways [180, 181]. Docosahexaenoic acid alleviates cancer aggressiveness by modulating the STAT3/nuclear factor kappa B (NF-kB) axis and M2 macrophage polarization [182, 183]. The outcomes of animal studies show that dietary n-3 PUFAs decrease proliferation and angiogenesis, while increasing apoptosis [184, 185]. An n-3 PUFA-enriched diet inhibits genomic DNA methylation, as well as Wnt, Akt, and mTOR signaling; this leads to suppressed cancer growth [186–189]. The beneficial effects of n-3 PUFAs on the risk of cancer have also been shown in human studies. Other cohort studies have revealed protective effects of n-3 PUFAs against breast, colon and endometrial cancer [190, 191].

The effect of MUFAs and n-6 PUFAs on the risk of development of cancer is inconsistent. Dietary n-6 PUFAs were significantly higher in malignant tissues and associated with prostate carcinogenesis [192]. On the other hand, a 2020 meta-analysis from all types of cancers showed n-6 PUFA were not related to carcinogenesis [193]. Another 2020 meta-analysis conducted by Kim et al. showed that intake of n-6 PUFAs was not significantly related with risk of cancer. However, blood levels of n-6 PUFAs were inversely associated with the risk of cancer [194]. Regarding MUFAs, OA treatment increases hypoxia-inducible factor-1 at the protein level and mediates cell survival, as well as colony and spheroid formation in HCC cells [195]. OA increases migration by regulating the PI3K/Akt pathway and NF-κB activity in breast cancer cells [196]. Additionally, a high olive oil diet promotes cell growth and metastasis of HeLa xenografts in mice [197]. In contrast, OA treatment decreases cell growth in breast cancer cells by suppressing HER-2 expression [198]. OA induces apoptosis and autophagy by inhibiting the Akt/mTOR pathway in tongue squamous cell carcinoma [199]. Li et al. reported that the various effects of OA on cell survival and migration result from AMPK dependency [200]. A meta-analysis of studies revealed a negative association between olive oil consumption and the risk of tumors [201]. Because olive oil contains several physiological substances with anti-inflammatory and antioxidant properties, an additional meta-analysis of cancer studies demonstrated a negative relationship between total MUFAs and the risk of cancer [202, 203]. In contrast, Liss et al. determined that MUFA consumption increases the risk of prostate cancer [204].

Dietary lipids, particularly n-3 PUFA supplements, improve clinical outcomes. When patients modulate the extent of lipid composition, they could be potential candidates for cancer therapy.

## Interplay between lipids and immune system in cancer cells

### Lipid-mediated modulation of immune system in cancer cells

The modulation of the immunity setting has been demonstrated to be an effective cancer treatment. Similar to cancers, the impact of immune responses depends on individual lipid composition. N-3 PUFAs have been shown antioxidant and anti-inflammatory properties by counteracting pro-inflammatory cytokines [205], whereas n-6 PUFAs exert pro-inflammatory effects in immune cells [206]. Excessive n-3 PUFAs in dendritic cells upregulate the expression of major histocompatibility complex class (MHC) class I like molecule, CD1d. The natural killer T cells recognize lipid antigens presented by CD1d, and n-3 PUFAs block optimal natural killer T cells activation and negatively affect the tumor progression [207]. Patients with a high-very long-chain FA consumption rate and lower serum very long-chain FA levels represent immunosuppressive tumor microenvironment [208]. Short-chain FAs recover an impaired immune response by increasing regulatory T cells frequency and affecting CD4(+) T cells and antigen-presenting cells [209]. Indeed, high levels of short-chain FA are associated with longer progression-free survival in patients with melanoma and lung cancer [210, 211]. In terms of CHO, cell receptor signaling is enhanced by increased CHO levels. An inhibitor of a CHO esterification enzyme increases the activities of CD8(+) T cells, thereby enhancing the efficacy of cancer immunotherapy based on programmed death 1 (PD-1) blockade [212].

Protein lipidation (e.g., myristoylation, prenylation and palmitoylation) can rewire the response of immune-related molecules that are responsible for cancer progression. For instance, a myristoyltransferase inhibitor prevents early B-cell receptor signaling events critical for cell survival. This inhibitor induces the degradation of the Src family and leads to the death of B-cell lymphoma cells [213]. RAS signaling in human cancer cells has anti-endoplasmic reticulum stress effect. Inhibition of RAS prenylation enhances endoplasmic reticulum stress and leads to the CD8(+) T cell-mediated cell death of colon cancer cells [214]. Palmitoylation stabilizes the programmed-death ligand 1 (PD-L1) by blocking its lysosomal degradation. The silencing of palmitoyl-transferase DHHC3 activates anticancer immunity of breast and colon cancer cells [215, 216]. Also, depletion of DHHC3 enhances recruitment of innate immune cells (antitumor M1-like macrophages and natural killer cells) and reduces the sizes of both the primary tumor and metastatic lung colonies [217]. Palmitoylation can stabilize interferon gamma receptor 1 and the MHC class I signaling pathways. In this way, palmitoylation can enhance T cell immunity and increase the sensitivity of colorectal cancer checkpoint treatment [218].

### Lipids and checkpoint inhibitors

Immune checkpoints such as PD-1, PDL-1 and cluster of differentiation 152 are molecules on immune cells that protect the immune system from cell death. Cancer cells use immune checkpoints to avoid being attacked by the immune system. Therefore, drugs targeting the immune environment of cancer have been applied. These drugs are called checkpoint inhibitors [219].

Recent studies suggest that lipid metabolism could be a modulator of anticancer immune responses. For instance, short chain FAs have beneficial effects on checkpoint inhibitors. Fecal and plasma samples from melanoma patients indicated that high levels of short chain FAs are associated with a positive response to PD-1 inhibitors (nivolumab and pembrolizumab) [210]. This result was also shown in a cohort study of patients with non-small cell lung cancer receiving PD-1 blockade [211]. Finally, the blood samples of patients with renal cell and urothelial carcinoma who were treated with checkpoint inhibitors (such as nivolumab, atezolizumab, and bevacizumab) were analyzed. Metabolomic analysis has shown that tumors with low levels of very long chain FA evade successful immune checkpoint inhibition [208].

The metabolic crosstalk between cancer cells and immune cells is a crucial determinant. Therefore, targeting lipid metabolism in the immune system will produce new therapies for cancer.

## Comparisons with other studies and what does the current review add to the existing knowledge

Most of the studies have focused on the expression patterns of lipids and their role in cancer. However, there is no comprehensive analysis to explore the role of transcription factors related to lipid metabolism in cancer cells. In this review, we discussed how transcription factors rewire cancer progression by regulating lipid metabolism.

### Study strengths and limitations

This review summarizes the role of transcription factors as a tumor regulator from the metabolic perspective, emphasizing that transcription factors could be potential targets for cancer therapy. We also have integrated the lipid-mediated regulation of cancer-immune interplay. However, this review has a limitation such as the lack of quantification of the altered levels of lipogenic enzymes. Because the levels of lipogenic enzymes are different between cancer tissues and patient’s sample types, comprehensive studies to analyze the profiles of lipogenic enzymes are necessary.

## Discussion

Cancer is one of the leading causes of mortality, and its incidence is closely related to metabolic change. Besides cancer-mediated metabolism, lipid metabolism is responsible for both carcinogenesis and cancer progression. In terms of start point of carcinogenesis, in vivo study and meta-analysis study from 3129 cancer patients showed that interference with lipid metabolism has the potential to decrease the cancer incidence [89, 163]. In both preclinical and clinical studies, genetic suppression or therapeutic administration targeting lipid metabolism revealed its importance in malignant phenotypes. Therefore, strategies targeting lipid metabolism will provide a new dimension to both preventive and therapeutic trials.

With all this information, cancer cells depend on transcription factors to support their growth and survival. High expression of transcription factors is associated with poor prognosis, and about 20% of oncogenes have been identified as transcription factors [19]. Besides lipid-mediated regulation, many transcription factors involved in carcinogenesis include inflammatory proteins, such as NF-kB, STAT3, and activator protein 1 [220–222]. Additionally, tumor microenvironmental proteins (e.g., hypoxia-inducible factors) contribute to oncogenesis by helping tumor cells survive hypoxia and nutrient deprivation [223]. As a nuclear receptor interacts with estrogen and progesterone, estrogen receptor-α activates signaling pathways of breast, ovarian, and prostate cancer [224]. Other well-established transcription factors in cancers are the epithelial-mesenchymal transition and proliferation markers. β-catenin/Wnt signaling stimulates the migration and activates downstream targets, such as cyclin D and the c-Myc transcription factors [225, 226]. The Myc family induces cell proliferation by activating the transcription of cyclin-dependent kinases and E2Fs [227, 228]. E2F is a family of transcription factors, including eight genes, such as E2F1–8, which a play critical role in regulating the G1-S phase of the cell cycle transition [229]. Many survival signals including PI3K/Akt, Wnt/Hedgehog, and MAPK pathways are closely associated with E2Fs, and hyper-activated E2Fs in tumors have been linked with a poor prognosis [230, 231].

Cancer studies revealed the crucial role of lipid metabolism in cancer progression. However, there are existing challenges: 1) the levels of lipids and lipid-related transcription factors vary between cancer types; 2) drugs have not been conclusive on the beneficial effect for patients. Drugs should be targeted carefully and more studies should be performed for the better management of cancers.

## Conclusion and future perspectives

Lipid metabolism has high potential as novel biomarkers for the diagnosis, prognosis, and therapy of cancer. Since the regulation of lipid metabolism is also an important gene for normal cells, it is still a huge challenge to find substances that target lipid metabolism and non-toxic effects of lipids. Fortunately, the quantitative determination of in vivo lipidomes and the cellular response to different growth conditions can be monitored in real-time using imaging modalities [232]. Additionally, current lipidomic approaches have identified more than 200,000 predicted lipid species [233]. Such efforts provide a route to a better understanding of disease biology and will be a promising strategy to treat cancer.

## Supplementary Information

**Additional file 1.**

## Data Availability

Not applicable.

## Abbreviations

ABCA1: Adenosine triphosphate binding cassette subfamily A member
ABCG1: Adenosine triphosphate binding cassette subfamily G member 1
ACC: Acetyl-CoA carboxylase
AMPK: Adenosine monophosphate-activated protein kinase
CHO: Cholesterol
ChREBP: Carbohydrate-response element-binding protein
ER: Endoplasmic reticulum
FAs: Fatty acids
FABPs: Fatty acid binding proteins
FASN: Fatty acid synthase
HCC: Hepatocellular carcinoma
HFD: High-fat diet
HMGCR: β-Hydroxy β-methylglutaryl-CoA reductase
LDL: Low-density lipoprotein
LXR: Liver X receptor
MHC: Major histocompatibility complex class
mTOR: Mammalian target of rapamycin
MUFAs: Monounsaturated fatty acids
NF-kB: Nuclear factor kappa B
OA: Oleic acid
PD-1: Programmed-death 1
PD-L1: Programmed-death ligand 1
PPAR: Peroxisome proliferator-activated receptor
PUFAs: Poly-unsaturated fatty acids
SCAP: SREBP cleavage activating protein
SCD1: Stearoyl-CoA desaturase1
SFAs: Saturated fatty acids
SREBPs: Sterol regulatory element-binding proteins
STAT3: Signal transducer and activator of transcription 3
TG: Triglycerides
