# Supplementary material for: How cancer cells remodel lipid metabolism: strategies targeting transcription factors
Source: Lipids Health Dis. 2021 Nov 14;20:163. doi: 10.1186/s12944-021-01593-8 (PMC8590761; doi:10.1186/s12944-021-01593-8)
Supplement: Supplementary file 1 — Additional file 1. [file 12944_2021_1593_MOESM1_ESM.pdf]

LHAD_manuscript

By Do-Won Jeong

WORD COUNT	15771	TIME SUBMITTED	05-OCT-2021 11:19AM
		PAPER ID	77357859

How cancer cells remodel lipid metabolism: strategies targeting transcription factors

Do-Won Jeong[1,2], Seulbee Lee[1,2], and Yang-Sook Chun[1,2,3*]

[1]Department of Biomedical Sciences, Seoul National University College of Medicine, Seoul 03080, Korea

[2]Department of Physiology, Seoul National University College of Medicine, Seoul 03080, Korea

[3]Ischemic/Hypoxic Disease Institute, Seoul National University College of Medicine, Seoul 03080, Korea

[*]Correspondence: chunys@snu.ac.kr

Abstract

Reprogramming of lipid metabolism has received increasing recognition as a hallmark of cancer cells because lipid dysregulation and the alteration of related enzyme profiles are closely correlated with oncogenic signals and malignant phenotypes, such as metastasis and therapeutic resistance. In this review, we describe recent findings that support the importance of lipids, as well as the transcription factors involved in cancer lipid metabolism. With recent advances in transcription factor analysis, including computer-modeling techniques, transcription factors are emerging as central players in cancer biology. Considering the limited number and the crucial role of transcription factors associated with lipid rewiring in cancers, transcription factor targeting is a promising potential strategy for cancer therapy.

Keywords: Lipid metabolism, Transcription factor, Cancer

Introduction

Cancer is a fundamental disorder of cell proliferation that requires many cellular building blocks, such as proteins, nucleic acids, and lipids. Cancer cells alter metabolism to accumulate metabolic intermediates as sources of these building blocks[1]. The best understood metabolic change is the Warburg effect, which involves exacerbation of glucose uptake and glycolysis[2]. Under normal conditions, glucose undergoes glycolysis to produce pyruvate. The tricarboxylic acid cycle and oxidative phosphorylation then extract energy in the form of adenosine triphosphate. However, in the absence of oxygen, glucose is metabolized to pyruvate, while excess glucose is converted to lactate. Cancer cells tend to prefer the Warburg effect even in the presence of oxygen, leading to increased glucose uptake and consumption, along with decreased oxidative phosphorylation[3]. Another commonly observed metabolic change is glutamine metabolism[4]. Glutamine consumption by cancer cells provides carbon and amino-nitrogen to synthesize nucleotides, amino acids, and lipids[5]. The third hallmark of cancers is altered lipid metabolism. Although changes in lipid metabolism have received less attention than other changes in cancer cells, recent studies have demonstrated a relationship between lipid reprogramming and cancer progression[6].

Lipids are a class of micro-biomolecules that encompass cholesterol (CHO), fatty acids (FAs), and their derivatives (e.g., mono-, di-, and triglycerides [TGs]). Lipids exert multiple biochemical functions in cells, such as membrane synthesis, energy production, and the activation of intracellular signaling pathways. For example, glycerol-lipids are stored lipids used for energy synthesis, while glycerophospholipids are used as structural components of cell membranes. Additionally, sphingolipids serve as signaling molecules to regulate various biological processes, such as cell growth, differentiation, and apoptosis[7]. Because highly proliferating cells require an aberrantly high supply of lipids, the changes in lipid composition and metabolism are considered hallmarks of cancer aggressiveness (e.g., breast, colon, liver and prostate cancers)[8]. Indeed, FA profiles and the levels of TGs and CHO change in the tissues and plasma of patients with various cancers[9-12]. Lipoproteins, such as low-density lipoprotein (LDL), are also higher in the plasma of cancer patients[13]. Importantly, cancer cells obtain the bulk of their lipids from *de novo* synthesis via transcription factor-dependent regulation, whereas most normal cells acquire their lipids from circulating exogenous lipids[14].

Transcription factors are responsible for the expression of genes needed to adapt to highly proliferating cellular conditions. Recent studies have demonstrated that several diseases are associated with changes in transcription factors. For example, Boyadjiev *et al.* reported that one-third of human developmental disorders and birth defects can be attributed to dysfunction and gene mutations encoding transcription factors[15]. Additionally, 164 transcription factors have been directly implicated in 277 diseases[16]. In cancers, 294 transcription factors have been identified by comparing a list of 1,571 candidate oncogenic proteins with a list of 1,988 human transcription factors and regulators[17, 18], which corresponds to approximately 19% of all known oncogenes[19]. In addition, fine-tuning of regulatory systems by transcription factors involved in lipid metabolism helps cancer cells adapt to the challenging microenvironment. For example, upregulation of lipogenic gene expression in cancers results from elevated expression and activation of sterol regulatory element-binding proteins (SREBPs)[20]. Excess carbohydrate intake and hyperglycemia caused by carbohydrate-response element-binding protein (ChREBP) lead to energy storage as TGs and promote tumor progression[21]. Although transcription factors are undruggable targets, an advanced understanding of transcription factors (including their structure) and the dynamics of binding to DNA can provide strategies for fighting cancers.

In this review, we focus on comprehensive insights into lipid metabolism in view of transcription factors, and highlight the complex interplay between lipids and immune system in cancer cells. We propose that lipids and lipid-related transcription factors have the potential to serve as effective therapeutic targets for anticancer immunotherapy.

Roles of lipids in cancer cells

1. Lipids in plasma membranes

Cell membranes contain hundreds of lipids and proteins; they are composed of sphingolipid-CHO-rich membrane rafts known as lipid rafts[22]. Rysman *et al*. analyzed cellular lipid extracts using a mass spectrometry-based approach and determined the tendency for lipid saturation in clinical tumor tissues, compared with normal tissues. Notably, lipid saturation results from lipid uptake toward *de novo* lipogenesis, combined with increases in saturated fatty acids (SFAs) and monounsaturated fatty acids (MUFAs), as well as a decrease in polyunsaturated fatty acids (PUFAs). SFAs are less prone to lipid peroxidation, compared with

PUFAs[23]. Additionally, SFAs reduce membrane fluidity, resulting in changes in death receptors, apoptotic stimuli, and metastasis in cancer cells[24]. Overall, these changes provide cancer cells with a survival benefit.

2. Lipids as signaling molecules

Lipids act as intra- and extracellular messengers; they can become potent mediators of malignant behavior. Sphingolipids are a class of lipids that contain ceramide and sphingosine, and they have multiple roles in cancer cell survival[7]. For example, sphingosine-1-phosphate, which is produced from sphingosine, increases cell proliferation and tumor malignancy by activating both signal transducer and activator of transcription 3 (STAT3) and the Warburg effect[25, 26]. Phosphoinositides are central mediators of the phosphoinositide 3-kinase (PI3K)/Akt/mammalian target of rapamycin (mTOR) signaling axis. PI3K activates the rapid conversion of phosphatidylinositol (PI; 4,5) P2 into PI(3,4,5)P3, which leads to the recruitment and activation of Akt[27]. PIP3 is also the substrate for phosphatase and tensin homolog (PTEN), and PTEN is most frequently mutated or deleted in cancer[28]. Increased levels of lysophosphatidic acid receptors have been described in several cancers; these increased levels contribute to cell invasiveness[29]. Lysophosphatidic acid is produced by the lysophospholipase autotaxin; it activates cell proliferation and tumor invasiveness by binding to G-protein-coupled receptors[30]. Prostaglandin E2 is an eicosanoid[31] that activates the Ras pathway and induces cell proliferation, which is associated with a poor prognosis[32]. Therefore, lipids are involved in multiple cellular signaling processes, many of which are linked to oncogenesis.

3. Lipids as protein modulators

Lipids regulate proteins by dynamic lipid post-translational modifications. Among these modifications, palmitoylation has attracted considerable interest, because it is essential for the functioning of key signaling oncoproteins[33]. For example, palmitoylation is required for Wnt secretion because it facilitates the interaction between Wnt and its intracellular chaperone Wntless[34]. The key oncoprotein Ras is also palmitoylated, thus facilitating membrane localization of Ras[35]. Epidermal growth factor receptor palmitoylation promotes PI3K/Akt signaling, leading to cell proliferation in lung cancer[36]. Another type of lipid-related post-translational modification is prenylation, in which the farnesyl group is covalently attached to target proteins[37]. Drugs targeting prenyltransferases have been used in preclinical trials; they

show protective effects in several solid cancers[38]. Guanosine triphosphate (GTP) enzymes (e.g., Cell division cycle 42, G-protein-coupled receptors, Ras, and Pho) may be prenylated, which is crucial for their membrane association and activation[39]. Indeed, the deletion of prenyltransferases inhibits the association between the Ras family and the plasma membrane; this delays Ras-induced lung tumor formation[40]. The role of lipids in cancers is illustrated in Fig. 1.

Lipid reprogramming in cancer

1. Changes in the lipid profile and cancer development

Recent advances in technologies including tandem mass spectrometry, RAMAN scattering microscopy, and electrospray ionization have enabled the quantitative analysis of lipids[41]. Differences in lipid profiles between malignant tumor specimens and matched normal tissues indicate that lipids are potential diagnostic biomarkers. For example, Qiu *et al.* investigated phospholipid composition in plasma samples of patients with hepatocellular carcinoma (HCC); they detected increases in SFAs and MUFAs. However, they found that PUFAs decrease in the plasma of HCC patients[42]. Integrated metabolomics and transcriptomics from 386 HCC cases showed that the levels of palmitic acid and oleic acid (OA) increase in patients with aggressive HCC[43]. A decrease in linoleic acid and an increase in OA were also found in patients with bladder cancer[44], lymphoma[45], and colorectal cancer[9]. An abundance of diverse lysophospholipids is present in colorectal cancer, compared with benign counterpart tissue[10]. Another study revealed reduced levels of TGs, stearic acid, myristic acid, palmitic acid, and OA in colorectal cancer patients; total phospholipids and PUFAs were increased in those patients[11]. Children with lymphoblastic leukemia have higher serum levels of TGs and LDL[13]. Similarly, plasma CHO and LDL are higher in breast cancer patients than in controls. In addition, breast cancer patients with lymphatic metastasis have higher CHO and TGs levels than do breast cancer patients without metastasis[46]. Thysell *et al.* demonstrated higher levels of CHO in prostate cancer patients with bone metastasis than in other types of cancer patients with bone metastasis from different tissues, as well as normal bone[12]. Changes of the lipid profile in cancers are presented in Table 1.

Polar lipids, multiple phospholipids, and other metabolites are also upregulated in prostate cancer cell lines[47]. Other studies have shown that high levels of SFAs and MUFAs are present in association with high metastatic potential in breast and melanoma cell lineages[48, 49]. Diglyceride is overexpressed in metastatic osteosarcoma cells; diglyceride targeting reduces cell migration, providing a clue that pharmacological targeting of certain lipids could be therapeutic[50]. Changes in the lipid profiles of various cancers are summarized in Table 1.

Table 1. Diagnostic lipid signatures in cancers

Cancer type	Control samples	Cancer samples	Biofluid	Altered lipids	References
Hepatocellular carcinoma	14	23	Plasma	SFA (12:0, 14:0, 15:0, 16:0, 20:0, 22:0)<br>MUFA (16:1n-7, 17:1n-9, 18:1n-9, 22:1n-9)<br>PUFA (18:2n-6, 20:4n-6, 22:5n-3)	[42]
	42	42	Tissue	SFA (14:0, 15:0)<br>MUFA (14:1, 16:1n-7, 17:1n-9, 18:1n-9, 24:1n-9)<br>PUFA (18:2n-6, 20:3n-6, 22:2n-6, 22:4n-6, 22:5n-3, 22:6n-3)	
	30	30	Tissue	SFA (4:0, 6:0, 7:0, 8:0, 16:0)<br>MUFA (16:1n-7, 20:1n-9)<br>PUFA (18:2n-6, 18:3n-3, 18:4n-3 20:1n-9, 20:2n-6, 20:5n-3, 22:2n-6, 22:3n-3, 22:5n-3, 22:6n-3), CHO	[43]

Bladder	31	31	Tissue	SFA (18:0)<br>MUFA (18:1n-9)<br>PUFA (20:4n-6)	[44]
Lymphoma	29	47	Plasma	SFA (16:0)<br>MUFA (18:1n-9)<br>PUFA (18:2n-6, 20:5n-3, 22:4n-6, 22:6n-3)	[45]
Colorectal cancer	12	17	Plasma	SFA (16:0)<br>MUFA (18:1n-7, 18:1n-9)<br>PUFA (18:2n-6, 20:3n-6)<br>TG, CHO	[9]
Colorectal cancer	20	20	Tissue	SFA (14:0, 18:0)<br>MUFA (16:1, 18:1)<br>PUFA (18:2, 20:4, 20:5, 22:2, 22:3)<br>TG, CHO	[10]
Colorectal cancer	25	25	Tissue	SFA (16:0, 18:0)<br>MUFA (16:1, 18:1)<br>PUFA (18:3, 20:4, 20:5, 22:6)<br>TG, CHO	[11]
Lymphoblastic leukemia	22	80	Plasma	TG, LDL	[13]
Breast cancer	60	120	Plasma	CHO, LDL	[46]
Prostate cancer	14	20	Plasma	SFA (18:0)<br>PUFA (18:2n-6, 20:4)<br>TG, CHO	[12]

SFA: saturated fatty acids; MUFA: monounsaturated fatty acids; PUFA: polyunsaturated fatty acids; TG: triglyceride; CHO: cholesterol; LDL: low-density lipoprotein; HDL: high-density lipoprotein

2. Abnormal levels of lipogenic enzymes in cancer

More than 90% of lipids required by cancer cells are reportedly derived from *de novo* synthesis. Consistent with these published findings, cancer cells often show upregulation of *de novo* lipogenic enzymes involved in the synthesis of FAs and CHO. For example, adenosine triphosphate citrate lyase and acetyl-coenzyme A carboxylase are upregulated and activated in most cancers[51]. Fatty acid synthase (FASN) is a metabolic oncogene, because elevated levels of FASN are detected in various solid tumors, including breast, prostate, colon, lung, bladder, ovary, stomach, endometrium, kidney, skin, esophagus, and tongue[52]. In addition, the upregulation of FASN is associated with unfavorable outcomes in patients with the most aggressive tumors. Abnormally elevated CHO levels in cancer cells result from 3-hydroxy-3-methylglutaryl coenzyme A reductase. High expression of 3-hydroxy-3-methylglutaryl coenzyme A reductase has been associated with poor pathological features and survival in breast cancer patients[53]. Overall, aberrantly high levels of lipids and lipogenic enzymes are significantly associated with the development, progression, and poor prognosis of several cancers.

Transcription factors leading to lipid metabolism rewiring in cancer

1. ChREBP

Glucose and other simple sugars obtained from the diet are metabolized to provide acetyl-CoA for the synthesis of FAs. During this process, ChREBP, encoded by the *MLXIPL* gene, acts as a transcriptional mediator to convert excess glucose to fat in the liver[54]. ChREBP binds to the carbohydrate-response elements of glycolytic and lipogenic genes, such as liver-type pyruvate kinase, *FASN*, and acetyl-coenzyme A carboxylase; through these interactions, ChREBP coordinates the carbohydrate induction of lipogenesis. Increased hepatic glucose accumulation and decreased hepatic FAs are detected in ChREBP knockout mice, compared with wild-type mice[55].

ChREBP has major roles in the pathogenesis of different types of cancer. ChREBP knockdown by siRNA significantly inhibits aerobic glycolysis and the synthesis of lipids in colon cancer cells. Additionally, ChREBP knockdown activates p53, induces cell cycle arrest, and reduces colon cancer growth *in vivo*, indicating an oncogenic function for ChREBP[56, 57]. Positive correlations of *MLXIPL* mRNA with glycolytic and lipogenic genes were also

observed in a comprehensive chromatin immunoprecipitation analysis of human HCC and breast cancer. Glucose transporter 1 increases significantly in HCC patients, and its expression is positively correlated with ChREBP. In contrast, the opposite association has been observed between the ChREBP protein and pyruvate dehydrogenase kinase 2 genes, which inactivate acetyl-CoA production[58]. Cancer cells favor the conversion of pyruvate into acetyl-CoA, rather than the accumulation of pyruvate[59]. Considering that the ChREBP protein is positively correlated with tumor malignancy[58], these results show that the oncogenic role of ChREBP/*MLXIPL* results from the conversion of glucose to fat[60].

2. FXR

Farnesoid X receptor (FXR), encoded by the *NR1H4* gene, was originally identified as a nuclear receptor activated by farnesol metabolites[61]. However, more recent studies have revealed that FXR is an endogenous bile acid-receptor that contributes to the maintenance of CHO/bile acid homeostasis by regulating various metabolic enzymes[62]. Because bile acids constitute a major factor that facilitates the absorption of dietary fats and steroids, the functions of FXR in metabolic diseases are well-established[63]. For example, FXR-null mice develop elevated serum CHO and TGs levels; however, they also show reduced adipocyte size and protective effects against high-fat diet (HFD)-induced obesity[64]. Genetic depletion of intestinal FXR in mice markedly decreases HFD-induced insulin resistance and fatty liver because of reductions in ceramide levels in the intestines and serum[65]. Although FXR-deficient mice are resistant to HFD-induced obesity, FXR agonists protect the liver from inflammation and fibrosis in the non-alcoholic steatohepatitis mouse model[66].

Similarly, FXR has crucial roles in the pathogenesis of several cancers. Loss of FXR function promotes intestinal fat absorption and synergistically increases liver carcinogenesis[67]. In mice, HFD-induced bile acid accumulation inhibits FXR function and subsequently leads to the progression of colorectal cancer. In contrast, the activation of intestinal FXR by a selective agonist restricts colon cell growth, regardless of HFD conditions[68]. Lipids isolated from bone induce the migration of breast cancer cells; FXR modulates this tumorigenic effect of bone-derived lipids, and eventually regulates the metastasis of breast tumor cells to bone[69]. Anakk *et al*. reported that loss of the FXR disrupts bile acid metabolism; it leads to activation of the Yes-associated protein and subsequent hepatocarcinogenesis[70]. Because studies of the FXR-mediated metabolic pathways have

mainly focused on bile acid signaling, the mechanisms regulated by FXR in cancers require further investigation.

3. LXRs

Liver X receptors (LXRs) have been an intriguing target for the treatment of inflammation, Alzheimer's disease, and cancer. LXRs are related to nuclear receptors (e.g., PPAR, FXR, and RXR) and are classified as a subfamily of *NR1H3* (LXRα) and *NR1H2* (LXRβ)[71]. LXRα is mainly expressed in liver tissue[72], whereas LXRβ is expressed in most tissues[73]. LXRs are upstream of the SREBP1c and ChREBP proteins; they directly regulate glycolysis and increase lipogenesis[74]. Compared with wild-type mice, LXR knockout mice show decreased levels of key lipogenic genes, including *SREBP1c*, *FASN*, and *SCD1*, as well as decreased production of TGs[75].

LXRs are CHO sensors that exert anticancer effects in various cancers. For example, LXR-driven genes, such as adenosine triphosphate binding cassette subfamily G member 1 (*ABCG1*) and apolipoprotein E, are expressed less frequently in breast cancer. The *ABCG1* and apolipoprotein E proteins are involved in CHO efflux from the cell to extracellular acceptors; the loss of LXR-mediated genes results in higher lipid contents in cells, as well as greater cancer cell viability[76]. Similarly, LXR agonists degrade the LDL receptor and increase the expression of the adenosine triphosphate binding cassette subfamily A member 1 (ABCA1) CHO efflux transporter, preventing exogenous CHO uptake in glioblastoma. Because glioblastoma cells require high levels of CHO for growth, LXR agonists promote tumor cell death[77]. Another LXR agonist induces increases in the *ABCG1* level and subsequently stimulates reverse CHO transport in prostate cancer cells. Atomic force microscopy scanning of the plasma membrane has revealed thinner lipid rafts after LXR stimulation. Thus, LXR agonists suppress the growth of prostate cancer cells in both xenografted nude mice and cell culture[78]. Furthermore, activation of LXRs decreases the expression of lipogenic genes (e.g., *SREBP1c*, *SCD1*, and *FASN*) in breast cancer cells. LXR agonists subsequently suppress cell cycle genes, indicating an anti-proliferative role for LXRs[79]. However, LXR antagonists inhibit both the Warburg effect and lipogenesis, thereby inducing apoptosis in colon, lung, and prostate cancer cells; notably, these antagonists have no toxic effects on non-malignant cells[80]. Considering that LXR agonists and antagonists show different effects, the

development of a strategy for LXRs (*NR1H3* and *NR1H2*) as an anticancer drug should be carefully considered.

4. PPARs

Peroxisome proliferator-activated receptors (PPARs) are transcription factors that control gene expression by binding to peroxisome proliferator response elements in the promoters of target genes[81, 82]. According to their tissue distribution and ligand specificity, PPARs are divided into three nuclear receptor subtypes: -α, -β/δ, and -γ[83]. PPARα is mainly expressed in tissues with high rates of FA β-oxidation, such as the liver, kidneys, heart, and muscle. PPARβ/δ is ubiquitously expressed in most tissues, and PPARγ is mainly expressed in adipose tissue[82, 84]. These PPAR isoforms modulate lipid metabolism in different manners. PPARα is a main energy-producing factor during nutrient deficiency and participates in FA β-oxidation. Additionally, PPARα downregulates hepatic apolipoprotein A-I and C-III; it also increases lipoprotein lipase gene expression, leading to a decrease in plasma TGs[85]. PPARβ/δ induces glucose 6-phosphate dehydrogenase activity, increases FA β-oxidation in muscle, and inhibits the release of FAs from white adipose tissue[86]. In diet-induced obese mice, the activation of PPARβ/δ normalizes serum insulin and TGs concentrations; it also acts as a new target for the treatment of type 2 diabetes[87]. Similarly, PPARβ/δ protects pancreatic islets against FA-induced β-cell dysfunction[88]. PPARγ promotes energy storage by directing FAs toward esterification and accumulation as TGs[89]. Dietary supplementation of the PPARγ agonist rosiglitazone suppresses type 2 diabetes in obese mice, but chronic treatment with rosiglitazone markedly aggravates hepatic steatosis[90].

In addition, clinically or preclinically relevant relationships between PPARs and cancers have been observed. Pan-cancer datasets of patients with 21 cancers show that altered PPARs signaling dysregulates numerous tumor cell lipid metabolic-related pathways to directly impact patient survival[91]. Moreover, mice fed a PPARα agonist exhibit lower body weights and an increased incidence of hepatic carcinogenesis. Tumors and visible nodules up to approximately 11 mm in diameter were apparent in livers from PPARα agonist-fed mice[92]. PPARδ induces xenograft tumor growth of prostate cancer cells by regulating the ATP binding cassette transporter 1 (*ABCA1)* gene. *ABCA1* is a CHO efflux-related gene; PPARδ directly increases the levels of *ABCA1* mRNA and membrane CHO expression, which is followed by tumor growth[93]. PPARγ has tumor-suppressive and oncogenic effects in several cancers. In lung

cancer cells, PPARγ-mediated lipid synthesis strongly induces mitochondrial reactive oxygen species stress and contributes to tumor suppression[94]. In addition, the chemical activation of PPARγ increases fatty acid binding protein 4 (FABP4) expression, which is accompanied by increased levels of intracellular reactive oxygen species in lung cancer cells. PPARγ-driven induction of FABP4 and lipoprotein lipase results in a better prognosis for lung and renal cancer patients[95]. In contrast, the interaction between PPARγ and Nur77 plays an antagonistic role in breast cancer. Nur77 recruits PPARγ to the CD36 promoter and FABP4 to suppress transcription of these genes, thus preventing FA uptake and cell proliferation. PPARγ physically binds to Nur77 and facilitates ubiquitin ligase Trim13-mediated ubiquitination of Nur77, thereby aggravating breast cancer[96].

Notably, dietary FAs directly bind to PPARs and mimic the effects of synthetic agonists that activate PPARs[97]. In this manner, PPARs sense the FA signals derived from dietary lipids and serve as lipid modulators to facilitate cancer progression. For example, an HFD promotes tumor cell growth and metastasis of colorectal cancer cells to the liver in mice. However, PPARδ deletion in mice completely inhibits the effect of an HFD, along with expression of Nanog and CD44, demonstrating that PPARs mediate the tumorigenic effect of HFD on cancer progression[98].

5. SREBPs

Lipid homeostasis is regulated by SREBP transcription factors. SREBPs directly activate the expression of more than 30 genes; they also contribute to the synthesis and uptake of CHO, FAs, and TGs. The N-terminal domain of SREBP must be proteolytically processed to act as a transcription factor. When cells experience CHO depletion, the SREBP cleavage activation protein (SCAP) escorts the SREBP from the endoplasmic reticulum to the Golgi apparatus. The SREBP is subsequently cleaved in the Golgi apparatus[99], and the mature SREBP translocates to the nucleus where it binds to a sterol response element in the promoter of the target gene[100]. The mammalian genome encodes three SREBP isoforms: SREBP1a, SREBP1c, and SREBP2[101]. The same gene on human chromosome 17p11.2 encodes SREBP1a and SREBP1c. However, SREBP2 is encoded by distinct genes on human chromosome 22q13[102]. SREBP1a is a potent activator of all SREBP-responsive genes, including genes involved in the synthesis of LDL receptor, CHO, and FAs; SREBP1c preferentially enhances the expression of

genes related to FA synthesis. Additionally, cells produce both SREBP1a and -2 to activate CHO synthesis in the presence of increased demands for CHO[100].

Recent studies have shown that SREBPs regulate lipid metabolism in cancers. Inhibition of SREBPs at the transcriptional level attenuates the expression of lipogenic genes and lipid uptake in patients with ovarian cancer. In this context, silencing of the SREBP genes abrogates ovarian tumor growth, blood vessel formation, and lipid content both *in vitro* and *in vivo*[103]. Consistent with these published findings, silencing of SREBP downstream genes showed that *de novo* FA synthesis and membrane phospholipids are required for breast and pancreatic cancer growth[24, 104]. SREBPs enhance the prenylation of N-Ras, leading to its activation and oncogenic effects[105].

Studies targeting the SREBP maturation pathways in cancers are well-established. Li *et al*. reported that pharmacological inhibition of SREBP proteolysis reduces HCC progression by regulating FA and CHO metabolism[106]. In various cancer cell lines, such as HeLa, T98, and U2OS, inhibition of SREBP-SCAP complex transport from the endoplasmic reticulum to the Golgi apparatus reveals anti-tumor properties. In addition to suppressing lipid metabolism, the inhibition of SREBP maturation perturbs tubulin polymerization and mitotic spindle assembly, leading to decreased cancer cell proliferation and migration[107].

SREBPs also provide survival advantages to cancer cells. Nuclear SREBP1 is correlated with high LDL receptor levels in glioblastoma patients. Because cancer cells use high levels of CHO for growth, SREBP-driven upregulation of the LDL receptor prevents apoptotic cell death[77]. In addition, SREBPs protect cancer cells from reactive oxygen species and endoplasmic reticulum stress by altering the ratio of SFAs to unsaturated long-chain FAs[108]. Because SFAs are less lipotoxic than PUFAs, SREBPs allow cancer cells to survive the harsh microenvironment[23]. These findings emphasize that SREBPs and their regulatory systems could serve as potential therapeutic targets. The role of lipid-related transcription factors in cancer is presented in Fig. 2.

Therapeutic strategies targeting lipid metabolism

Due to the central role of lipids and related transcription factors in cancer, continuous efforts have been made to adjust lipid metabolism as anticancer drugs. This approach was employed in preclinical models both *in vitro* and *in vivo*, and some of the drugs have entered clinical trials.

Interestingly, lipids themselves showed promising effects on cancer. Here, we listed a brief overview of therapeutic drugs targeting transcription factors and lipid-related enzymes and lipids.

1. Drugs targeting FXR

GW4064 is a synthetic FXR agonist that increases cell proliferation and invasion by activating NF-κB and N-cadherin in HCC and pancreatic cancer cells[109, 110]. However, some drugs that target FXR have shown a protective effect on tumors. GW4064 inhibits proliferation and migration and induces apoptosis in colon cancer, esophageal cancer and liver cancer[111-113]. The FXR antagonist, guggulsterone, inhibits migration in liver cancer and pancreatic cancer by regulating NF-kB[109, 110]. Since the effectiveness of drugs targeting FXR varies with the type of cancer tissue, treatments should be designed for specific organs.

2. Drugs targeting LXR

The LXR inhibitor, SR9243, inhibits the Warburg effect and lipogenesis, and induces apoptosis of cancer cells, but is not toxic to non-malignant cells[80]. Pommier *et al*. The elucidated LXR activator T0901317 down-regulates the Akt pathway and induces apoptosis *in vivo* and in prostate cancer cells[78]. The LXR agonist GW3965 induces cell death by degrading LDL receptors and increasing the expression of ABCA1 CHO efflux transporter[77]. Another LXR agonist, LXR-623, crosses the blood-brain barrier and reaches therapeutic levels in glioblastoma cells[114].

3. Drugs targeting PPARs

The PPARα agonist, fenofibrate, inhibits tumor growth and angiogenesis in melanoma and fibrosarcoma[115]. In breast cancer, fenofibrate promotes chemotherapy sensitivity by down-regulating Mcl-1 and Bcl-xl and up-regulating Bok and Bax at the transcriptional level[116]. Fenofibrate reduces the migration of oral cancer cells and promotes the autophagy of prostate tumors *in vivo* by interfering with the Warburg effect and regulating the adenosine monophosphate-activated protein kinase (AMPK)-mTOR pathway[117, 118]. In addition, as a selective PPARα ligand, Wy-14643 down-regulates cytochrome P450 CYP2C, an enzyme that catalyzes the epoxidation of PUFA, and also inhibits endothelial cell proliferation and tumorigenesis[119, 120]. However, chronic treatment of Wy-14643 increases the incidence of liver cancer through induction of oxidative stress *in vivo*[92].

Genetic suppression of PPARδ inhibits tumor growth of prostate cancer cells[93], however, some of the studies have reported the anticancer activity of PPARβ/δ agonists. Zaveri *et al.* suggested that PPARβ/δ antagonist, SR13904, inhibits the proliferation of the lung cancer cell[121]. PPARβ/δ agonist, GW0742, can improve the efficacy of preventing skin tumors, especially when combined with cyclooxygenase 2 inhibitors[122, 123].

Thiazolidinedione (also known as glitazones) is newly developed synthetic ligand of PPARγ[124]. Mechanistically, thiazolidinedione directly binds and leads to conformational change in PPARγ, and activates the transcriptional machinery. PPARγ agonists are used clinically as antidiabetic agents, resulting in sensitizing insulin and lowering blood glucose concentration[125]. Besides the metabolic actions, thiazolidinedione leads to cell death by targeting cyclin-dependent kinase in malignant cell lines (e.g., colon, liver and pancreatic cancer)[126-130]. Thiazolidinedione decreases the release of endothelial growth factor from smooth muscle cells and inhibits angiogenesis in glioblastoma, liposarcoma, lung and prostate cancer[131, 132]. Thiazolidinedione also prevents metastasis formation by forcing invasive cancer cells into adipogenesis[133]. PPARγ antagonists, T0070907 and GW9662, have protective effects on the breast and bladder cancer cells[134, 135]. However, drugs targeting PPARs have not been conclusive on the beneficial effect for patients[136, 137]. Clinical trials need more research.

4. Drugs targeting SREBPs

Silibinin is isolated from the seeds of the milk thistle plants, and mechanistically increases SREBP1 phosphorylation and inhibits its nuclear translocation. In breast cancer cells, silibinin promotes autophagy by inhibiting estrogen receptor-α and upregulating estrogen receptor-β[138]. Silibinin inhibits cell invasion by decreasing the production of urokinase-plasminogen activator and matrix metalloproteinase-2 in human lung cancer cells[139]. Silibinin also showed strong effect on head and neck and prostate cancer[140-142].

Betulin, fatostatin and nelfinavir suppress the processing of SREBPs and have anti-tumor properties. Li *et al*. found that betulin dramatically reduces diethylnitrosamine-induced HCC progression by downregulating tumor promoting cytokines such as interleukin-6 and -1β[106]. Fatostatin inhibits cell proliferation and invasion both *in vitro* and *in vivo* (e.g., glioblastoma, osteosarcoma, breast and prostate cancer)[107, 143, 144]. Compound 24 is a derivative of fatostatin, which was developed to improve chemical properties in a variety of disease

models[145]. Compound 24 decreases the levels of FAs and cellular growth in glioma, prostate, and breast cancer cell lines[108]. Nelvinavir was approved by the Food and Drug Administration for HIV treatment in humans at first, and is currently in phase II clinical trials for myeloma, glioblastoma, pancreatic and lung cancer. *In vitro*, nelfinavir inhibits cell proliferation in liposarcoma and prostate cancer cells[146, 147].

5. Drugs targeting lipid metabolism

FASN inhibitors have been the subject of many studies and one of the drugs that have entered clinical trials is TVB-2640. TVB-2640 showed significant anti-cancer activities across multiple tumor types including lung, ovarian, and breast cancer[148]. C75, another compound targeting FASN, directly increases FA oxidation and results in apoptosis during S phase in breast cancer cells[149, 150]. Cerulenin reduces the transactivation of estrogen receptors in ovarian cancer, and suppresses liver metastasis of colon cancer, and induces cancer cell death both *in vivo* and *in vitro*[151-153]. Orlistat is a drug approved for obesity management by the Food and Drug Administration in 1999. Orlistat induces endoplasmic reticulum stress and subsequently increases apoptosis in breast, colon and prostate cancer cells[154, 155].

Preclinical investigations are ongoing on the use of an inhibitor of ACC. Soraphen A induces cell growth arrest and cytotoxicity in prostate cancer cells[156]. ND-646 is an allosteric inhibitor of ACC, which inhibits the growth of non-small cell lung tumor growth *in vitro* and *in vivo*[157]. Another allosteric inhibitor of ACC, TOFA, induces cell death by releasing proapoptotic proteins from mitochondria in breast, colon, lung and ovarian cancer[158-160]. SCD1 inhibitor A939572 induces endoplasmic reticulum stress and enhances cell death in clear cell renal cell carcinoma[161]. A939572 decreases the phosphorylation of the PI3K/Akt pathway and significantly suppresses the cell vitality of lung cancer cells *in vivo*[162]. Blockage of SCD1 with CAY10566 induces cell apoptosis by promoting ceramide synthesis in colon cancer and inactivating the AMPK signaling in HCC[163, 164]. CAY10566 also showed protective effect on glioblastoma, melanoma, ovarian and lung cancer[165-167].

Targeting CHO synthesis could be another potential approach. Statin is a HMGCR inhibitor and has shown promising outcomes *in vitro* and *in vivo*. Statin inhibits lipid metabolism and leads to cell survival in various cancers (e.g., colon, pancreatic, liver, breast, prostate, bladder, lung and skin cancer)[168, 169]. In 3,129 human epidemiologic studies, the use of statin reduces incidence and recurrence of various cancers (e.g., bladder, breast, colon, kidney, lung,

skin, pancreas and prostate cancer)[170-173]. Another CHO synthesis inhibitor simvastatin also inhibits the Akt pathway and induces apoptosis in prostate cancer cells[174]. However, a recent meta-analysis of cancers revealed no significant effect of statin on cancer therapy indicating that the clinical use of drugs targeting CHO should be carefully considered[175, 176].

The challenge of targeting lipid uptake is focused on CD36 and FABPs. ABT-510 is a synthetic analog of thrombospondin-1 and reaches phase I clinical studies against glioblastoma, melanoma and renal cell carcinoma[177-179]. Mechanistically, ABT-510 directly binds to CD36 and induces both Fas and Fas-L expression[180, 181]. dmrFABP5 is synthesized by switching three amino acids of FABP5, losing its ability to bind FAs. dmrFABP5 significantly suppresses proliferation, migration, and invasion of prostate cancer cells[182]. SBFI-102 and SBFI-103 increase cytotoxicity in prostate cancer cells and stimulate tumor-suppressive effects of other chemicals, taxanes[183]. EI-05, FABP5 activator, enhances lipid droplet and interferon-β production, which further promotes the anti-tumor activity of macrophages during inflammation. In breast cancer cells, administration of EI-05 *in vivo* significantly inhibits cell growth[184]. The list of lipid-related drugs is summarized in Table 2.

Table 2. List of lipid-related drugs

Target	Drug	Phase of development	Cancer type	Reference
FXR	GW4064	Preclinical	Colon, esophagus, liver and pancreatic cancer	[109-113]
	guggulsterone	Preclinical	Liver and pancreatic cancer	[109, 110]
LXR	GW3965	Preclinical	Glioblastoma	[77]
	LXR-623	Phase I	Glioblastoma	[114]
	SR9243	Preclinical	Colon cancer	[80]
	T0901317	Preclinical	Prostate cancer	[78]
PPARα	Fenofibrate	Preclinical	Breast, oral and prostate cancer, Melanoma	[115-118]
	Wy-14643	Preclinical	Breast, colon, lung and liver cancer	[92, 119]
PPARβ/δ	SR13904	Preclinical	Lung cancer	[121]
	GW0742	Preclinical	Melanoma	[122, 123]

PPARγ	Thiazolidinedione	Phase III	Colon, pancreatic, liver and breast cancer, Liposarcoma	[126-131, 134]
	T0070907	Preclinical	Breast cancer	[134]
	GW9662	Preclinical	Bladder cancer	[135]
SREBPs	Sibilinin	Clinical	Breast, head and neck, lung, and prostate cancer	[138-142]
	Betulin	Preclinical	HCC	[106]
	Fatostatin	Preclinical	Glioblastoma, osteosarcoma, breast and prostate cancer	[107, 143, 144]
	Compound 24	Preclinical	Glioblastoma	[108]
S1P/S2P	Nelfinavir	Phase II	Glioblastoma, liposarcoma, myeloma, lung, pancreatic, and prostate cancer	[146, 147, 185]
FASN	TVB-2640	Phase II	Lung, ovarian and breast cancer	[148]
	C75	Preclinical	Breast, colon, ovarian, and prostate cancer	[149, 150, 153, 154, 186]
	Cerulenin	Preclinical	Colon and ovarian cancer	[151-153]
	Orlistat	Approved for anti-obesity	Breast, colon and prostate cancer	[154, 155]
ACC	Soraphen A	Preclinical	Prostate cancer	[156]
	ND-646	Preclinical	Lung cancer	[157]
	TOFA	Preclinical	Breast, colon, lung, and prostate cancer	[158-160]
SCD	A939572	Preclinical	Clear cell renal cell carcinoma, Lung cancer	[161, 162]
	CAY10566	Preclinical	Colon and ovarian cancer, glioblastoma, melanoma, HCC	[163-167]

HMGCR	Statin	Approved	Many cancers	[168-173, 175, 176]
	Simvastatin	Preclinical	Prostate cancer	[174]
CD36	ABT-510	Phase I	Glioblastoma, melanoma and renal cell carcinoma	[177-179]
FABP5	dmrFABP5	Preclinical	Prostate cancer	[182]
	SBFI-102, SBFI-103	Preclinical	Prostate cancer	[183]
	EI-05	Preclinical	Breast cancer	[184]

FXR: Farnesoid X receptor; SREBP: Sterol regulatory element-binding protein; S1P: site-1 protease; LXR: Liver X receptor; ChREBP: Carbohydrate-response element-binding protein; PPARs: Peroxisome proliferator-activated receptor; FASN: Fatty acid synthase; ACC: Acetyl-coenzyme A carboxylase; SCD: Stearoyl-CoA desaturase; HCC: Hepatocellular carcinoma; HMGCR: HMG-CoA reductase

Dietary lipids and cancer

Because excess calorie intake and obesity are linked to a high risk of cancer aggressiveness, many studies have focused on dietary adjustments as a potential target for cancer therapy[187]. An HFD has been regarded as a common pathogenic factor in many diseases. However, recent studies have identified that the effect of dietary fat depends on the composition of individual FAs. SFAs are positively associated with carcinogenesis, whereas n-3 PUFAs are more likely to have protective effects against cancer[188, 189]. Data regarding the effects of MUFAs and n-6 PUFA on cancer progression are controversial.

The protective effects of n-3 PUFAs have been confirmed in numerous cancer cell lines. Docosahexaenoic acid is a major n-3 PUFA involved in anticancer activity. *In vitro* studies have demonstrated that docosahexaenoic acid inhibits cancer cell proliferation and resistance to irradiation by regulating the Akt and Wnt pathways[190, 191]. Docosahexaenoic acid alleviates cancer aggressiveness by modulating the STAT3/nuclear factor kappa B (NF-kB) axis and M2 macrophage polarization[192, 193]. The outcomes of animal studies show that dietary n-3 PUFAs decrease proliferation and angiogenesis, while increasing apoptosis[194, 195]. An n-3 PUFA-enriched diet inhibits genomic DNA methylation, as well as Wnt, Akt, and mTOR

signaling; this leads to suppressed cancer growth[196-199]. The beneficial effects of n-3 PUFAs on the risk of cancer have also been shown in human studies. Other cohort studies have revealed protective effects of n-3 PUFAs against breast, colon and endometrial cancer[200, 201].

The effect of MUFAs and n-6 PUFAs on the risk of development of cancer is inconsistent. Dietary n-6 PUFAs were significantly higher in malignant tissues and associated with prostate carcinogenesis[202]. On the other hand, a 2020 meta-analysis from all types of cancers showed n-6 PUFA were not related to carcinogenesis[203]. Another 2020 meta-analysis conducted by Kim *et al.* showed that intake of n-6 PUFAs was not significantly related with risk of cancer. However, blood levels of n-6 PUFAs were inversely associated with the risk of cancer[204]. Regarding MUFAs, OA treatment increases hypoxia-inducible factor-1 at the protein level and mediates cell survival, as well as colony and spheroid formation in HCC cells[205]. OA increases migration by regulating the PI3K/Akt pathway and NF-κB activity in breast cancer cells[206]. Additionally, a high olive oil diet promotes cell growth and metastasis of HeLa xenografts in mice[207]. In contrast, OA treatment decreases cell growth in breast cancer cells by suppressing HER-2 expression[208]. OA induces apoptosis and autophagy by inhibiting the Akt/mTOR pathway in tongue squamous cell carcinoma[209]. Li *et al.* reported that the various effects of OA on cell survival and migration result from AMPK dependency[210]. A meta-analysis of studies revealed a negative association between olive oil consumption and the risk of tumors[211]. Because olive oil contains several physiological substances with anti-inflammatory and antioxidant properties, an additional meta-analysis of cancer studies demonstrated a negative relationship between total MUFAs and the risk of cancer[212, 213]. In contrast, Liss *et al.* determined that MUFA consumption increases the risk of prostate cancer[214].

Dietary lipids, particularly n-3 PUFA supplements, improve clinical outcomes. When patients modulate the extent of lipid composition, they could be potential candidates for cancer therapy.

Interplay between lipids and immune system in cancer cells

1. Lipid-mediated modulation of immune system in cancer cells

The modulation of the immunity setting has been demonstrated to be an effective cancer

treatment. Similar to cancers, the impact of immune responses depends on individual lipid composition. N-3 PUFAs have been shown antioxidant and anti-inflammatory properties by counteracting pro-inflammatory cytokines[215], whereas n-6 PUFAs exert pro-inflammatory effects in immune cells[216]. Excessive n-3 PUFAs in dendritic cells upregulate the expression of major histocompatibility complex class (MHC) class I like molecule, CD1d. The natural killer T cells recognize lipid antigens presented by CD1d, and n-3 PUFAs block optimal natural killer T cells activation and negatively affect the tumor progression[217]. Immunosuppressive type II tumor-associated macrophages and T cells uptake energy from the FA oxidation rather than glycolysis. Inhibition of lipid oxidation prevents immunosuppressive capabilities of regulatory T cells and enhances the survival of mice with glioblastoma[218]. Likewise, CD8(+) tumor infiltrating lymphocytes reprogram the metabolic phenotypes to FA catabolism through PPARα signaling, and delay melanoma growth[219]. Patients with a high-very long-chain FA consumption rate and lower serum very long-chain FA levels represent immunosuppressive tumor microenvironment[220]. Short-chain FAs recover an impaired immune response by increasing regulatory T cells frequency and affecting CD4(+) T cells and antigen-presenting cells[221]. Indeed, high levels of short-chain FA are associated with longer progression-free survival in patients with melanoma and lung cancer[222, 223].

In terms of CHO, cell receptor signaling is enhanced by increased CHO levels. An inhibitor of a CHO esterification enzyme increases the activities of CD8(+) T cells, thereby enhancing the efficacy of cancer immunotherapy based on programmed death 1 (PD-1) blockade[224]. On the other hand, Ma *et al.* suggested that CHO induces CD8(+) T cell exhaustion in the tumor microenvironment and helps cancer cells to survive. T cell exhaustion is characterized by CD8+ T cell dysfunction, exhausted CD8(+) T cells have a high expression of inhibitory T cell receptors and natural killer cell receptors. Tumor-specific CD8(+) T cells uptake CHO and become exhausted T cells in metastatic lung tumor model and human colon cancer[225].

Protein lipidation (e.g., myristoylation, prenylation and palmitoylation) can rewire the response of immune-related molecules that are responsible for cancer progression. For instance, a myristoyltransferase inhibitor prevents early B-cell receptor signaling events critical for cell survival. This inhibitor induces the degradation of the Src family and leads to the death of B-cell lymphoma cells[226]. RAS signaling in human cancer cells has anti-endoplasmic reticulum stress effect. Inhibition of RAS prenylation enhances endoplasmic reticulum stress

and leads to the CD8(+) T cell-mediated cell death of colon cancer cells[227]. Palmitoylation stabilizes the programmed-death ligand 1 (PD-L1) by blocking its lysosomal degradation. The silencing of palmitoyl-transferase DHHC3 activates anticancer immunity of breast and colon cancer cells[228, 229]. Also, depletion of DHHC3 enhances recruitment of innate immune cells (antitumor M1-like macrophages and natural killer cells) and reduces the sizes of both the primary tumor and metastatic lung colonies[230]. Palmitoylation can stabilize interferon gamma receptor 1 and the MHC class I signaling pathways. In this way, palmitoylation can enhance T cell immunity and increase the sensitivity of colorectal cancer checkpoint treatment[231].

2. Lipids and checkpoint inhibitors

Immune checkpoints such as PD-1, PDL-1 and cluster of differentiation 152 are molecules on immune cells that protect the immune system from cell death. Cancer cells use immune checkpoints to avoid being attacked by the immune system. Therefore, drugs targeting the immune environment of cancer have been applied. These drugs are called checkpoint inhibitors[232].

Recent studies suggest that lipid metabolism could be a modulator of anticancer immune responses. For instance, short chain FAs have beneficial effects on checkpoint inhibitors. Fecal and plasma samples from melanoma patients indicated that high levels of short chain FAs are associated with a positive response to PD-1 inhibitors (nivolumab and pembrolizumab)[222]. This result was also shown in a cohort study of patients with non-small cell lung cancer receiving PD-1 blockade[223]. Finally, the blood samples of patients with renal cell and urothelial carcinoma who were treated with checkpoint inhibitors (such as nivolumab, atezolizumab, and bevacizumab) were analyzed. Metabolomic analysis has shown that tumors with low levels of very long chain FA evade successful immune checkpoint inhibition[220].

The metabolic crosstalk between cancer cells and immune cells is a crucial determinant. Therefore, targeting lipid metabolism in the immune system will produce new therapies for cancer.

Comparisons with other studies and what does the current review add to the existing knowledge

Most of the studies have focused on the expression patterns of lipids and their role in cancer. However, there is no comprehensive analysis to explore the role of transcription factors related to lipid metabolism in cancer cells. In this review, we discussed how transcription factors rewire cancer progression by regulating lipid metabolism.

Study strengths and limitations

This review summarizes the role of transcription factors as a tumor regulator from the metabolic perspective, emphasizing that transcription factors could be potential targets for cancer therapy. We also have integrated the lipid-mediated regulation of cancer-immune interplay. However, this review has a limitation such as the lack of quantification of the altered levels of lipogenic enzymes. Because the levels of lipogenic enzymes are different between cancer tissues and patient's sample types, comprehensive studies to analyze the profiles of lipogenic enzymes are necessary.

Conclusion and future perspectives

Cancer is one of the leading causes of mortality, and its incidence is closely related to metabolic change. Besides cancer-mediated metabolism, lipid metabolism is responsible for both carcinogenesis and cancer progression. In terms of start point of carcinogenesis, *in vivo* study and meta-analysis study from 3,129 cancer patients showed that interference with lipid metabolism has the potential to decrease the cancer incidence[92, 173]. In both preclinical and clinical studies, genetic suppression or therapeutic administration targeting lipid metabolism revealed its importance in malignant phenotypes. Therefore, strategies targeting lipid metabolism will provide a new dimension to both preventive and therapeutic trials.

Since the regulation of lipid metabolism is also an important gene for normal cells, it is still a huge challenge to find substances that target lipid metabolism and non-toxic effects of lipids. Fortunately, the quantitative determination of *in vivo* lipidomes and the cellular response to different growth conditions can be monitored in real-time using imaging modalities[233]. Additionally, current lipidomic approaches have identified more than 200,000 predicted lipid species[234]. Such efforts provide a route to a better understanding of disease biology.

With all this information, cancer cells depend on transcription factors to support their growth and survival. High expression of transcription factors is associated with poor prognosis, and

about 20% of oncogenes have been identified as transcription factors[19]. Many transcription factors involved in carcinogenesis include inflammatory proteins, such as NF-kB, STAT3, and activator protein 1[235-237]. Additionally, tumor microenvironmental proteins (e.g., hypoxia-inducible factors) contribute to oncogenesis by helping tumor cells survive hypoxia and nutrient deprivation[238]. As a nuclear receptor interacts with estrogen and progesterone, estrogen receptor-α activates signaling pathways of breast, ovarian, and prostate cancer[239]. Other well-established transcription factors in cancers are the epithelial-mesenchymal transition and proliferation markers. β-catenin/Wnt signaling stimulates the migration and activates downstream targets, such as cyclin D and the c-Myc transcription factors[240, 241]. The Myc family induces cell proliferation by activating the transcription of cyclin-dependent kinases and E2Fs[242, 243]. E2F is a family of transcription factors, including eight genes, such as E2F1–8, which a play critical role in regulating the G1-S phase of the cell cycle transition[244]. Many survival signals including PI3K/Akt, Wnt/Hedgehog, and MAPK pathways are closely associated with E2Fs, and hyper-activated E2Fs in tumors have been linked with a poor prognosis[245, 246].

In summary, lipid metabolism has high potential as novel biomarkers for the diagnosis, prognosis, and therapy of cancer. However, the levels of lipids and lipid-related transcription factors vary between cancer types and some drugs have not been conclusive on the beneficial effect for patients. Drugs should be targeted carefully and more studies should be performed for the better management of cancers.

List of abbreviations

ABCA1: adenosine triphosphate binding cassette subfamily A member; ABCG1: adenosine triphosphate binding cassette subfamily G member 1; AMPK: Adenosine monophosphate-activated protein kinase; CHO: Cholesterol; ChREBP: Carbohydrate-response element-binding protein; ER: Endoplasmic reticulum; FAs: Fatty acids; FABPs: Fatty acid binding proteins; FASN: Fatty acid synthase; HCC: Hepatocellular carcinoma; HFD: High-fat diet; LDL: Low-density lipoprotein; LXR: Liver X receptor; MHC: Major histocompatibility complex class; mTOR: Mammalian target of rapamycin; MUFAs: Monounsaturated fatty acids; NF-kB: Nuclear factor kappa B; OA: Oleic acid; PD-1: programmed-death 1; PD-L1: Programmed-death ligand 1; PPAR: Peroxisome proliferator-activated receptor; PUFAs: Poly-

unsaturated fatty acids; SCAP: SREBP cleavage activating protein; SFAs: Saturated fatty acids; SREBPs: Sterol regulatory element-binding proteins; STAT3: Signal transducer and activator of transcription 3; TG: Triglycerides.

Declarations

Ethics approval and consent to participate

Not applicable.

Consent for publication

Not applicable.

Availability of data and materials

Not applicable.

Competing interests

The authors declare that they have no competing interests.

Funding

This work was supported by National Research Foundation of Korea (No. 2019R1A2C2083886, 2018R1A5A2025964).

Authors' contributions

DWJ designed the outline, and wrote the manuscript. SL wrote and revised the manuscript. YSC designed the concept, wrote, reviewed and supervised the manuscript. All authors read and approved the final manuscript.

Acknowledgements

DWJ and SL received a scholarship from the BK21-plus program, Republic of Korea.

Figure legends

Fig. 1 Diagrammatic sketch of the role of lipids in cancers.

Fig. 2 A summary of the role of lipid-related transcription factors in cancer cells.

References

1. Hanahan D, Weinberg RA: **Hallmarks of cancer: the next generation**. *Cell* 2011, **144**(5):646-674.
2. Warburg O: **On the origin of cancer cells**. *Science* 1956, **123**(3191):309-314.

3. Liberti MV, Locasale JW: **The Warburg Effect: How Does it Benefit Cancer Cells?** *Trends Biochem Sci* 2016, **41**(3):211-218.

4. DeBerardinis RJ, Mancuso A, Daikhin E, Nissim I, Yudkoff M, Wehrli S, Thompson CB: **Beyond aerobic glycolysis: transformed cells can engage in glutamine metabolism that exceeds the requirement for protein and nucleotide synthesis**. *Proc Natl Acad Sci U S A* 2007, **104**(49):19345-19350.

5. Cluntun AA, Lukey MJ, Cerione RA, Locasale JW: **Glutamine Metabolism in Cancer: Understanding the Heterogeneity**. *Trends Cancer* 2017, **3**(3):169-180.

6. Patterson AD, Maurhofer O, Beyoglu D, Lanz C, Krausz KW, Pabst T, Gonzalez FJ, Dufour JF, Idle JR: **Aberrant lipid metabolism in hepatocellular carcinoma revealed by plasma metabolomics and lipid profiling**. *Cancer Res* 2011, **71**(21):6590-6600.

7. Ohotski J, Edwards J, Elsberger B, Watson C, Orange C, Mallon E, Pyne S, Pyne NJ: **Identification of novel functional and spatial associations between sphingosine kinase 1, sphingosine 1-phosphate receptors and other signaling proteins that affect prognostic outcome in estrogen receptor-positive breast cancer**. *Int J Cancer* 2013, **132**(3):605-616.

8. Baenke F, Peck B, Miess H, Schulze A: **Hooked on fat: the role of lipid synthesis in cancer metabolism and tumour development**. *Dis Model Mech* 2013, **6**(6):1353-1363.

9. Baro L, Hermoso JC, Nunez MC, Jimenez-Rios JA, Gil A: **Abnormalities in plasma and red blood cell fatty acid profiles of patients with colorectal cancer**. *Br J Cancer* 1998, **77**(11):1978-1983.

10. Wang Y, Hinz S, Uckermann O, Honscheid P, von Schonfels W, Burmeister G, Hendricks A, Ackerman JM, Baretton GB, Hampe J *et al*: **Shotgun lipidomics-based characterization of the landscape of lipid metabolism in colorectal cancer**. *Biochim Biophys Acta Mol Cell Biol Lipids* 2020, **1865**(3):158579.

11. Mika A, Pakiet A, Czumaj A, Kaczynski Z, Liakh I, Kobiela J, Perdyan A, Adrych K, Makarewicz W, Sledzinski T: **Decreased Triacylglycerol Content and Elevated Contents of Cell Membrane Lipids in Colorectal Cancer Tissue: A Lipidomic Study**. *J Clin Med* 2020, **9**(4).

12. Thysell E, Surowiec I, Hornberg E, Crnalic S, Widmark A, Johansson AI, Stattin P, Bergh A, Moritz T, Antti H *et al*: **Metabolomic characterization of human prostate cancer bone metastases reveals increased levels of cholesterol**. *PLoS One* 2010, **5**(12):e14175.

13. Morel S, Leahy J, Fournier M, Lamarche B, Garofalo C, Grimard G, Poulain F, Delvin E, Laverdiere C, Krajinovic M *et al*: **Lipid and lipoprotein abnormalities in acute lymphoblastic leukemia survivors**. *J Lipid Res* 2017, **58**(5):982-993.

14. Beloribi-Djefaflia S, Vasseur S, Guillaumond F: **Lipid metabolic reprogramming in cancer cells**. *Oncogenesis* 2016, **5**:e189.

15. Boyadjiev SA, Jabs EW: **Online Mendelian Inheritance in Man (OMIM) as a knowledgebase for human developmental disorders**. *Clin Genet* 2000, **57**(4):253-266.

16. Vaquerizas JM, Kummerfeld SK, Teichmann SA, Luscombe NM: **A census of human transcription factors: function, expression and evolution**. *Nat Rev Genet* 2009, **10**(4):252-263.

17. An O, Dall'Olio GM, Mourikis TP, Ciccarelli FD: **NCG 5.0: updates of a manually curated repository of cancer genes and associated properties from cancer mutational screenings**. *Nucleic Acids Res* 2016, **44**(D1):D992-999.

18. Ravasi T, Suzuki H, Cannistraci CV, Katayama S, Bajic VB, Tan K, Akalin A, Schmeier S, Kanamori-Katayama M, Bertin N *et al*: **An atlas of combinatorial transcriptional regulation in mouse and man**. *Cell* 2010, **140**(5):744-752.

19. Lambert M, Jambon S, Depauw S, David-Cordonnier MH: **Targeting Transcription Factors for Cancer Treatment**. *Molecules* 2018, **23**(6).

20. Yahagi N, Shimano H, Hasegawa K, Ohashi K, Matsuzaka T, Najima Y, Sekiya M, Tomita S, Okazaki H, Tamura Y *et al*: **Co-ordinate activation of lipogenic enzymes in hepatocellular carcinoma**. *Eur J Cancer* 2005, **41**(9):1316-1322.

21. Ryu TY, Park J, Scherer PE: **Hyperglycemia as a risk factor for cancer progression**. *Diabetes Metab J* 2014, **38**(5):330-336.

22. van Meer G, Voelker DR, Feigenson GW: **Membrane lipids: where they are and how they behave**. *Nat Rev Mol Cell Biol* 2008, **9**(2):112-124.

23. Rysman E, Brusselmans K, Scheys K, Timmermans L, Derua R, Munck S, Van Veldhoven PP, Waltregny D, Daniels VW, Machiels J *et al*: **De novo lipogenesis protects cancer cells from free radicals and chemotherapeutics by promoting membrane lipid saturation**. *Cancer Res* 2010, **70**(20):8117-8126.

24. Hilvo M, Denkert C, Lehtinen L, Muller B, Brockmoller S, Seppanen-Laakso T, Budczies J, Bucher E, Yetukuri L, Castillo S *et al*: **Novel theranostic opportunities offered by characterization of altered membrane lipid metabolism in breast cancer progression**. *Cancer Res* 2011, **71**(9):3236-3245.

25. Liang J, Nagahashi M, Kim EY, Harikumar KB, Yamada A, Huang WC, Hait NC, Allegood JC, Price MM, Avni D *et al*: **Sphingosine-1-phosphate links persistent STAT3 activation, chronic intestinal inflammation, and development of colitis-associated cancer**. *Cancer Cell* 2013, **23**(1):107-120.

26. Watson DG, Tonelli F, Alossaimi M, Williamson L, Chan E, Gorshkova I, Berdyshev E, Bittman R, Pyne NJ, Pyne S: **The roles of sphingosine kinases 1 and 2 in regulating the Warburg effect in prostate cancer cells**. *Cell Signal* 2013, **25**(4):1011-1017.

27. Thapa N, Tan X, Choi S, Lambert PF, Rapraeger AC, Anderson RA: **The Hidden Conundrum of Phosphoinositide Signaling in Cancer**. *Trends Cancer* 2016, **2**(7):378-390.

28. Li J, Yen C, Liaw D, Podsypanina K, Bose S, Wang SI, Puc J, Miliaresis C, Rodgers L, McCombie R *et al*: **PTEN, a putative protein tyrosine phosphatase gene mutated in human brain, breast, and prostate cancer**. *Science* 1997, **275**(5308):1943-1947.

29. Park SY, Jeong KJ, Panupinthu N, Yu S, Lee J, Han JW, Kim JM, Lee JS, Kang J, Park CG *et al*: **Lysophosphatidic acid augments human hepatocellular carcinoma cell invasion through LPA1 receptor and MMP-9 expression**. *Oncogene* 2011, **30**(11):1351-1359.

30. Benesch MG, Ko YM, McMullen TP, Brindley DN: **Autotaxin in the crosshairs: taking aim at cancer and other inflammatory conditions**. *FEBS Lett* 2014, **588**(16):2712-2727.

31. Krysan K, Reckamp KL, Dalwadi H, Sharma S, Rozengurt E, Dohadwala M, Dubinett SM: **Prostaglandin E2 activates mitogen-activated protein kinase/Erk pathway**

signaling and cell proliferation in non-small cell lung cancer cells in an epidermal growth factor receptor-independent manner. *Cancer Res* 2005, **65**(14):6275-6281.

32. Greenhough A, Smartt HJ, Moore AE, Roberts HR, Williams AC, Paraskeva C, Kaidi A: **The COX-2/PGE2 pathway: key roles in the hallmarks of cancer and adaptation to the tumour microenvironment**. *Carcinogenesis* 2009, **30**(3):377-386.
33. Tabaczar S, Czogalla A, Podkalicka J, Biernatowska A, Sikorski AF: **Protein palmitoylation: Palmitoyltransferases and their specificity**. *Exp Biol Med* 2017, **242**(11):1150-1157.
34. Takada R, Satomi Y, Kurata T, Ueno N, Norioka S, Kondoh H, Takao T, Takada S: **Monounsaturated fatty acid modification of Wnt protein: its role in Wnt secretion**. *Dev Cell* 2006, **11**(6):791-801.
35. Taguchi T, Misaki R: **Palmitoylation pilots ras to recycling endosomes**. *Small GTPases* 2011, **2**(2):82-84.
36. Runkle KB, Kharbanda A, Stypulkowski E, Cao XJ, Wang W, Garcia BA, Witze ES: **Inhibition of DHHC20-Mediated EGFR Palmitoylation Creates a Dependence on EGFR Signaling**. *Mol Cell* 2016, **62**(3):385-396.
37. Wang M, Casey PJ: **Protein prenylation: unique fats make their mark on biology**. *Nat Rev Mol Cell Biol* 2016, **17**(2):110-122.
38. Berndt N, Hamilton AD, Sebti SM: **Targeting protein prenylation for cancer therapy**. *Nat Rev Cancer* 2011, **11**(11):775-791.
39. Sebti SM: **Protein farnesylation: implications for normal physiology, malignant transformation, and cancer therapy**. *Cancer Cell* 2005, **7**(4):297-300.
40. Sjogren AK, Andersson KM, Liu M, Cutts BA, Karlsson C, Wahlstrom AM, Dalin M, Weinbaum C, Casey PJ, Tarkowski A *et al*: **GGTase-I deficiency reduces tumor formation and improves survival in mice with K-RAS-induced lung cancer**. *J Clin Invest* 2007, **117**(5):1294-1304.
41. Loizides-Mangold U: **On the future of mass-spectrometry-based lipidomics**. *FEBS J* 2013, **280**(12):2817-2829.
42. Qiu JF, Zhang KL, Zhang XJ, Hu YJ, Li P, Shang CZ, Wan JB: **Abnormalities in Plasma Phospholipid Fatty Acid Profiles of Patients with Hepatocellular Carcinoma**. *Lipids* 2015, **50**(10):977-985.

43. Budhu A, Roessler S, Zhao X, Yu Z, Forgues M, Ji J, Karoly E, Qin LX, Ye QH, Jia HL *et al*: **Integrated metabolite and gene expression profiles identify lipid biomarkers associated with progression of hepatocellular carcinoma and patient outcomes**. *Gastroenterology* 2013, **144**(5):1066-1075.e1.

44. Miryaghoubzadeh J, Darabi M, Madaen K, Shaaker M, Mehdizadeh A, Hajihosseini R: **Tissue fatty acid composition in human urothelial carcinoma**. *Br J Biomed Sci* 2013, **70**(1):1-5.

45. Cvetkovic Z, Vucic V, Cvetkovic B, Petrovic M, Ristic-Medic D, Tepsic J, Glibetic M: **Abnormal fatty acid distribution of the serum phospholipids of patients with non-Hodgkin lymphoma**. *Ann Hematol* 2010, **89**(8):775-782.

46. Abdelsalam KE, Hassan IK, Sadig IA: **The role of developing breast cancer in alteration of serum lipid profile**. *J Res Med Sci* 2012, **17**(6):562-5.

47. Sorvina A, Bader CA, Caporale C, Carter EA, Johnson IRD, Parkinson-Lawrence EJ, Simpson PV, Wright PJ, Stagni S, Lay PA *et al*: **Lipid profiles of prostate cancer cells**. *Oncotarget* 2018, **9**(85):35541-35552.

48. Kim HY, Lee KM, Kim SH, Kwon YJ, Chun YJ, Choi HK: **Comparative metabolic and lipidomic profiling of human breast cancer cells with different metastatic potentials**. *Oncotarget* 2016, **7**(41):67111-67128.

49. Kim HY, Lee H, Kim SH, Jin H, Bae J, Choi HK: **Discovery of potential biomarkers in human melanoma cells with different metastatic potential by metabolic and lipidomic profiling**. *Sci Rep* 2017, **7**(1):8864.

50. Roy J, Dibaeinia P, Fan TM, Sinha S, Das A: **Global analysis of osteosarcoma lipidomes reveal altered lipid profiles in metastatic versus nonmetastatic cells**. *J Lipid Res* 2019, **60**(2):375-387.

51. Menendez JA, Lupu R: **Fatty acid synthase and the lipogenic phenotype in cancer pathogenesis**. *Nat Rev Cancer* 2007, **7**(10):763-777.

52. Migita T, Ruiz S, Fornari A, Fiorentino M, Priolo C, Zadra G, Inazuka F, Grisanzio C, Palescandolo E, Shin E *et al*: **Fatty acid synthase: a metabolic enzyme and candidate oncogene in prostate cancer**. *J Natl Cancer Inst* 2009, **101**(7):519-532.

53. Gustbee E, Tryggvadottir H, Markkula A, Simonsson M, Nodin B, Jirstrom K, Rose C, Ingvar C, Borgquist S, Jernstrom H: **Tumor-specific expression of HMG-CoA**

reductase in a population-based cohort of breast cancer patients. *BMC Clin Pathol* 2015, **15**:8.

54. Herman MA, Peroni OD, Villoria J, Schon MR, Abumrad NA, Bluher M, Klein S, Kahn BB: **A novel ChREBP isoform in adipose tissue regulates systemic glucose metabolism**. *Nature* 2012, **484**(7394):333-338.
55. Iizuka K, Bruick RK, Liang G, Horton JD, Uyeda K: **Deficiency of carbohydrate response element-binding protein (ChREBP) reduces lipogenesis as well as glycolysis**. *Proc Natl Acad Sci U S A* 2004, **101**(19):7281-7286.
56. Lei Y, Zhou S, Hu Q, Chen X, Gu J: **Carbohydrate response element binding protein (ChREBP) correlates with colon cancer progression and contributes to cell proliferation**. *Sci Rep* 2020, **10**(1):4233.
57. Tong X, Zhao F, Mancuso A, Gruber JJ, Thompson CB: **The glucose-responsive transcription factor ChREBP contributes to glucose-dependent anabolic synthesis and cell proliferation**. *Proc Natl Acad Sci U S A* 2009, **106**(51):21660-21665.
58. Lei Y, Hu Q, Gu J: **Expressions of Carbohydrate Response Element Binding Protein and Glucose Transporters in Liver Cancer and Clinical Significance**. *Pathol Oncol Res* 2020, **26**(2):1331-1340.
59. Contractor T, Harris CR: **p53 negatively regulates transcription of the pyruvate dehydrogenase kinase Pdk2**. *Cancer Res* 2012, **72**(2):560-567.
60. Airley RE, McHugh P, Evans AR, Harris B, Winchester L, Buffa FM, Al-Tameemi W, Leek R, Harris AL: **Role of carbohydrate response element-binding protein (ChREBP) in generating an aerobic metabolic phenotype and in breast cancer progression**. *Br J Cancer* 2014, **110**(3):715-723.
61. Deaton AM, Sulem P, Nioi P, Benonisdottir S, Ward LD, Davidsson OB, Lao S, Helgadottir A, Fan F, Jensson BO *et al*: **A rare missense variant in NR1H4 associates with lower cholesterol levels**. *Commun Biol* 2018, **1**:14.
62. Matsubara T, Li F, Gonzalez FJ: **FXR signaling in the enterohepatic system**. *Mol Cell Endocrinol* 2013, **368**(1-2):17-29.
63. Qi Y, Jiang C, Cheng J, Krausz KW, Li T, Ferrell JM, Gonzalez FJ, Chiang JY: **Bile acid signaling in lipid metabolism: metabolomic and lipidomic analysis of lipid**

and bile acid markers linked to anti-obesity and anti-diabetes in mice. *Biochim Biophys Acta* 2015, **1851**(1):19-29.

64. Cariou B, van Harmelen K, Duran-Sandoval D, van Dijk TH, Grefhorst A, Abdelkarim M, Caron S, Torpier G, Fruchart JC, Gonzalez FJ *et al*: **The farnesoid X receptor modulates adiposity and peripheral insulin sensitivity in mice**. *J Biol Chem* 2006, **281**(16):11039-11049.

65. Gonzalez FJ, Jiang C, Patterson AD: **An Intestinal Microbiota-Farnesoid X Receptor Axis Modulates Metabolic Disease**. *Gastroenterology* 2016, **151**(5):845-859.

66. Zhang S, Wang J, Liu Q, Harnish DC: **Farnesoid X receptor agonist WAY-362450 attenuates liver inflammation and fibrosis in murine model of non-alcoholic steatohepatitis**. *J Hepatol* 2009, **51**(2):380-388.

67. Zhang Y, Ge X, Heemstra LA, Chen WD, Xu J, Smith JL, Ma H, Kasim N, Edwards PA, Novak CM: **Loss of FXR protects against diet-induced obesity and accelerates liver carcinogenesis in ob/ob mice**. *Mol Endocrinol* 2012, **26**(2):272-280.

68. Modica S, Murzilli S, Salvatore L, Schmidt DR, Moschetta A: **Nuclear bile acid receptor FXR protects against intestinal tumorigenesis**. *Cancer Res* 2008, **68**(23):9589-9594.

69. Silva J, Dasgupta S, Wang G, Krishnamurthy K, Ritter E, Bieberich E: **Lipids isolated from bone induce the migration of human breast cancer cells**. *J Lipid Res* 2006, **47**(4):724-733.

70. Anakk S, Bhosale M, Schmidt VA, Johnson RL, Finegold MJ, Moore DD: **Bile Acids Activate YAP to Promote Liver Carcinogenesis**. *Cell Rep* 2013, **5**(4):1060-1069.

71. Patel MB, Oza NA, Anand IS, Deshpande SS, Patel CN: **Liver x receptor: a novel therapeutic target**. *Indian J Pharm Sci* 2008, **70**(2):135-144.

72. Kim KH, Lee GY, Kim JI, Ham M, Won Lee J, Kim JB: **Inhibitory effect of LXR activation on cell proliferation and cell cycle progression through lipogenic activity**. *J Lipid Res* 2010, **51**(12):3425-3433.

73. Korach-Andre M, Gustafsson JA: **Liver X receptors as regulators of metabolism**. *Biomol Concepts* 2015, **6**(3):177-190.

74. Bindesboll C, Fan Q, Norgaard RC, MacPherson L, Ruan HB, Wu J, Pedersen TA, Steffensen KR, Yang X, Matthews J *et al*: **Liver X receptor regulates hepatic nuclear**

O-GlcNAc signaling and carbohydrate responsive element-binding protein activity. *J Lipid Res* 2015, **56**(4):771-785.

75. Steffensen KR, Gustafsson JA: **Liver X receptors: new drug targets to treat Type 2 diabetes?** *Future Lipidol* 2006, **1**(2):181-189.

76. Bobin-Dubigeon C, Chauvin A, Brillaud-Meflah V, Boiffard F, Joalland MP, Bard JM: **Liver X Receptor (LXR)-regulated Genes of Cholesterol Trafficking and Breast Cancer Severity**. *Anticancer Res* 2017, **37**(10):5495-5498.

77. Guo D, Reinitz F, Youssef M, Hong C, Nathanson D, Akhavan D, Kuga D, Amzajerdi AN, Soto H, Zhu S *et al*: **An LXR agonist promotes glioblastoma cell death through inhibition of an EGFR/AKT/SREBP-1/LDLR-dependent pathway**. *Cancer Discov* 2011, **1**(5):442-456.

78. Pommier AJ, Alves G, Viennois E, Bernard S, Communal Y, Sion B, Marceau G, Damon C, Mouzat K, Caira F *et al*: **Liver X Receptor activation downregulates AKT survival signaling in lipid rafts and induces apoptosis of prostate cancer cells**. *Oncogene* 2010, **29**(18):2712-2723.

79. Vedin LL, Lewandowski SA, Parini P, Gustafsson JA, Steffensen KR: **The oxysterol receptor LXR inhibits proliferation of human breast cancer cells**. *Carcinogenesis* 2009, **30**(4):575-579.

80. Flaveny CA, Griffett K, El-Gendy Bel D, Kazantzis M, Sengupta M, Amelio AL, Chatterjee A, Walker J, Solt LA, Kamenecka TM *et al*: **Broad Anti-tumor Activity of a Small Molecule that Selectively Targets the Warburg Effect and Lipogenesis**. *Cancer Cell* 2015, **28**(1):42-56.

81. Grygiel-Gorniak B: **Peroxisome proliferator-activated receptors and their ligands: nutritional and clinical implications - a review**. *Nutr J* 2014, **13**.

82. Varga T, Czimmerer Z, Nagy L: **PPARs are a unique set of fatty acid regulated transcription factors controlling both lipid metabolism and inflammation**. *Biochim Biophys Acta* 2011, **1812**(8):1007-1022.

83. Schoonjans K, Staels B, Auwerx J: **Role of the peroxisome proliferator-activated receptor (PPAR) in mediating the effects of fibrates and fatty acids on gene expression**. *J Lipid Res* 1996, **37**(5):907-925.

84. Feige JN, Gelman L, Michalik L, Desvergne B, Wahli W: **From molecular action to physiological outputs: peroxisome proliferator-activated receptors are nuclear receptors at the crossroads of key cellular functions**. *Prog Lipid Res* 2006, **45**(2):120-159.
85. Peters JM, Hennuyer N, Staels B, Fruchart JC, Fievet C, Gonzalez FJ, Auwerx J: **Alterations in lipoprotein metabolism in peroxisome proliferator-activated receptor alpha-deficient mice**. *J Biol Chem* 1997, **272**(43):27307-27312.
86. Wang YP, Nakajima T, Gonzalez FJ, Tanaka N: **PPARs as Metabolic Regulators in the Liver: Lessons from Liver-Specific PPAR-Null Mice**. *International Journal of Molecular Sciences* 2020, **21**(6).
87. Luquet S, Gaudel C, Holst D, Lopez-Soriano J, Jehl-Pietri C, Fredenrich A, Grimaldi PA: **Roles of PPAR delta in lipid absorption and metabolism: a new target for the treatment of type 2 diabetes**. *Biochim Biophys Acta* 2005, **1740**(2):313-317.
88. Ravnskjaer K, Frigerio F, Boergesen M, Nielsen T, Maechler P, Mandrup S: **PPARdelta is a fatty acid sensor that enhances mitochondrial oxidation in insulin-secreting cells and protects against fatty acid-induced dysfunction**. *J Lipid Res* 2010, **51**(6):1370-1379.
89. Ravnskjaer K, Boergesen M, Rubi B, Larsen JK, Nielsen T, Fridriksson J, Maechler P, Mandrup S: **Peroxisome proliferator-activated receptor alpha (PPARalpha) potentiates, whereas PPARgamma attenuates, glucose-stimulated insulin secretion in pancreatic beta-cells**. *Endocrinology* 2005, **146**(8):3266-3276.
90. Watkins SM, Reifsnyder PR, Pan HJ, German JB, Leiter EH: **Lipid metabolome-wide effects of the PPARgamma agonist rosiglitazone**. *J Lipid Res* 2002, **43**(11):1809-1817.
91. Chang WH, Lai AG: **The pan-cancer mutational landscape of the PPAR pathway reveals universal patterns of dysregulated metabolism and interactions with tumor immunity and hypoxia**. *Ann N Y Acad Sci* 2019, **1448**(1):65-82.
92. Peters JM, Cattley RC, Gonzalez FJ: **Role of PPAR alpha in the mechanism of action of the nongenotoxic carcinogen and peroxisome proliferator Wy-14,643**. *Carcinogenesis* 1997, **18**(11):2029-2033.

93. Her NG, Jeong SI, Cho K, Ha TK, Han J, Ko KP, Park SK, Lee JH, Lee MG, Ryu BK *et al*: **PPARdelta promotes oncogenic redirection of TGF-beta1 signaling through the activation of the ABCA1-Cav1 pathway**. *Cell Cycle* 2013, **12**(10):1521-1535.

94. Phan ANH, Vo VTA, Hua TNM, Kim MK, Jo SY, Choi JW, Kim HW, Son J, Suh YA, Jeong Y: **PPARgamma sumoylation-mediated lipid accumulation in lung cancer**. *Oncotarget* 2017, **8**(47):82491-82505.

95. Hua TNM, Kim MK, Vo VTA, Choi JW, Choi JH, Kim HW, Cha SK, Park KS, Jeong Y: **Inhibition of oncogenic Src induces FABP4-mediated lipolysis via PPARgamma activation exerting cancer growth suppression**. *EBioMedicine* 2019, **41**:134-145.

96. Yang PB, Hou PP, Liu FY, Hong WB, Chen HZ, Sun XY, Li P, Zhang Y, Ju CY, Luo LJ *et al*: **Blocking PPARgamma interaction facilitates Nur77 interdiction of fatty acid uptake and suppresses breast cancer progression**. *Proc Natl Acad Sci U S A* 2020, **117**(44):27412-27422.

97. Kliewer SA, Sundseth SS, Jones SA, Brown PJ, Wisely GB, Koble CS, Devchand P, Wahli W, Willson TM, Lenhard JM *et al*: **Fatty acids and eicosanoids regulate gene expression through direct interactions with peroxisome proliferator-activated receptors alpha and gamma**. *P Natl Acad Sci USA* 1997, **94**(9):4318-4323.

98. Wang D, Fu L, Wei J, Xiong Y, DuBois RN: **PPARdelta Mediates the Effect of Dietary Fat in Promoting Colorectal Cancer Metastasis**. *Cancer Res* 2019, **79**(17):4480-4490.

99. Horton JD: **Sterol regulatory element-binding proteins: transcriptional activators of lipid synthesis**. *Biochem Soc Trans* 2002, **30**(Pt 6):1091-1095.

100. Brown MS, Goldstein JL: **The SREBP pathway: regulation of cholesterol metabolism by proteolysis of a membrane-bound transcription factor**. *Cell* 1997, **89**(3):331-340.

101. Lee JH, Phelan P, Shin M, Oh BC, Han X, Im SS, Osborne TF: **SREBP-1a-stimulated lipid synthesis is required for macrophage phagocytosis downstream of TLR4-directed mTORC1**. *Proc Natl Acad Sci U S A* 2018, **115**(52):E12228-E12234.

102. Xu X, So JS, Park JG, Lee AH: **Transcriptional control of hepatic lipid metabolism by SREBP and ChREBP**. *Semin Liver Dis* 2013, **33**(4):301-311.

103. Chakraborty PK, Xiong X, Mustafi SB, Saha S, Dhanasekaran D, Mandal NA, McMeekin S, Bhattacharya R, Mukherjee P: **Role of cystathionine beta synthase in lipid metabolism in ovarian cancer**. *Oncotarget* 2015, **6**(35):37367-37384.
104. Sun Y, He W, Luo M, Zhou Y, Chang G, Ren W, Wu K, Li X, Shen J, Zhao X *et al*: **SREBP1 regulates tumorigenesis and prognosis of pancreatic cancer through targeting lipid metabolism**. *Tumour Biol* 2015, **36**(6):4133-4141.
105. Shamma A, Takegami Y, Miki T, Kitajima S, Noda M, Obara T, Okamoto T, Takahashi C: **Rb Regulates DNA damage response and cellular senescence through E2F-dependent suppression of N-ras isoprenylation**. *Cancer Cell* 2009, **15**(4):255-269.
106. Li N, Zhou ZS, Shen Y, Xu J, Miao HH, Xiong Y, Xu F, Li BL, Luo J, Song BL: **Inhibition of the sterol regulatory element-binding protein pathway suppresses hepatocellular carcinoma by repressing inflammation in mice**. *Hepatology* 2017, **65**(6):1936-1947.
107. Gholkar AA, Cheung K, Williams KJ, Lo YC, Hamideh SA, Nnebe C, Khuu C, Bensinger SJ, Torres JZ: **Fatostatin Inhibits Cancer Cell Proliferation by Affecting Mitotic Microtubule Spindle Assembly and Cell Division**. *J Biol Chem* 2016, **291**(33):17001-17008.
108. Williams KJ, Argus JP, Zhu Y, Wilks MQ, Marbois BN, York AG, Kidani Y, Pourzia AL, Akhavan D, Lisiero DN *et al*: **An essential requirement for the SCAP/SREBP signaling axis to protect cancer cells from lipotoxicity**. *Cancer Res* 2013, **73**(9):2850-2862.
109. Kainuma M, Takada I, Makishima M, Sano K: **Farnesoid X Receptor Activation Enhances Transforming Growth Factor beta-Induced Epithelial-Mesenchymal Transition in Hepatocellular Carcinoma Cells**. *Int J Mol Sci* 2018, **19**(7).
110. Lee JY, Lee KT, Lee JK, Lee KH, Jang KT, Heo JS, Choi SH, Kim Y, Rhee JC: **Farnesoid X receptor, overexpressed in pancreatic cancer with lymph node metastasis promotes cell migration and invasion**. *Br J Cancer* 2011, **104**(6):1027-1037.
111. Guo F, Xu Z, Zhang Y, Jiang P, Huang G, Chen S, Lyu X, Zheng P, Zhao X, Zeng Y *et al*: **FXR induces SOCS3 and suppresses hepatocellular carcinoma**. *Oncotarget* 2015, **6**(33):34606-34616.

112. Feng Q, Zhang H, Yao D, Zhang X, Chen WD, Wang YD: **Activation of FXR Suppresses Esophageal Squamous Cell Carcinoma Through Antagonizing ERK1/2 Signaling Pathway**. *Cancer Manag Res* 2021, **13**:5907-5918.

113. Mao J, Chen X, Wang C, Li W, Li J: **Effects and mechanism of the bile acid (farnesoid X) receptor on the Wnt/beta-catenin signaling pathway in colon cancer**. *Oncol Lett* 2020, **20**(1):337-345.

114. Villa GR, Hulce JJ, Zanca C, Bi J, Ikegami S, Cahill GL, Gu Y, Lum KM, Masui K, Yang H *et al*: **An LXR-Cholesterol Axis Creates a Metabolic Co-Dependency for Brain Cancers**. *Cancer Cell* 2016, **30**(5):683-693.

115. Panigrahy D, Kaipainen A, Huang S, Butterfield CE, Barnes CM, Fannon M, Laforme AM, Chaponis DM, Folkman J, Kieran MW: **PPARalpha agonist fenofibrate suppresses tumor growth through direct and indirect angiogenesis inhibition**. *Proc Natl Acad Sci U S A* 2008, **105**(3):985-990.

116. Sun J, Zheng Z, Chen Q, Pan Y, Quan M, Dai Y: **Fenofibrate potentiates chemosensitivity to human breast cancer cells by modulating apoptosis via AKT/NF-kappaB pathway**. *Onco Targets Ther* 2019, **12**:773-783.

117. Huang YP, Chang NW: **PPARalpha modulates gene expression profiles of mitochondrial energy metabolism in oral tumorigenesis**. *Biomedicine (Taipei)* 2016, **6**(1):3.

118. Tao T, Zhao F, Xuan Q, Shen Z, Xiao J, Shen Q: **Fenofibrate inhibits the growth of prostate cancer through regulating autophagy and endoplasmic reticulum stress**. *Biochem Biophys Res Commun* 2018, **503**(4):2685-2689.

119. Pozzi A, Ibanez MR, Gatica AE, Yang S, Wei S, Mei S, Falck JR, Capdevila JH: **Peroxisomal proliferator-activated receptor-alpha-dependent inhibition of endothelial cell proliferation and tumorigenesis**. *J Biol Chem* 2007, **282**(24):17685-17695.

120. Aoyama T, Peters JM, Iritani N, Nakajima T, Furihata K, Hashimoto T, Gonzalez FJ: **Altered constitutive expression of fatty acid-metabolizing enzymes in mice lacking the peroxisome proliferator-activated receptor alpha (PPARalpha)**. *J Biol Chem* 1998, **273**(10):5678-5684.

121. Zaveri NT, Sato BG, Jiang F, Calaoagan J, Laderoute KR, Murphy BJ: **A novel peroxisome proliferator-activated receptor delta antagonist, SR13904, has anti-proliferative activity in human cancer cells**. *Cancer Biol Ther* 2009, **8**(13):1252-1261.

122. Zhu B, Bai R, Kennett MJ, Kang BH, Gonzalez FJ, Peters JM: **Chemoprevention of chemically induced skin tumorigenesis by ligand activation of peroxisome proliferator-activated receptor-beta/delta and inhibition of cyclooxygenase 2**. *Mol Cancer Ther* 2010, **9**(12):3267-3277.

123. Bility MT, Zhu B, Kang BH, Gonzalez FJ, Peters JM: **Ligand activation of peroxisome proliferator-activated receptor-beta/delta and inhibition of cyclooxygenase-2 enhances inhibition of skin tumorigenesis**. *Toxicol Sci* 2010, **113**(1):27-36.

124. Peters AL: **Using thiazolidinediones: rosiglitazone and pioglitazone in clinical practice**. *Am J Manag Care* 2001, **7**(3 Suppl):S87-95; quiz S96-87.

125. Berger J, Bailey P, Biswas C, Cullinan CA, Doebber TW, Hayes NS, Saperstein R, Smith RG, Leibowitz MD: **Thiazolidinediones produce a conformational change in peroxisomal proliferator-activated receptor-gamma: binding and activation correlate with antidiabetic actions in db/db mice**. *Endocrinology* 1996, **137**(10):4189-4195.

126. Shimada T, Kojima K, Yoshiura K, Hiraishi H, Terano A: **Characteristics of the peroxisome proliferator activated receptor gamma (PPARgamma) ligand induced apoptosis in colon cancer cells**. *Gut* 2002, **50**(5):658-664.

127. Eibl G, Wente MN, Reber HA, Hines OJ: **Peroxisome proliferator-activated receptor gamma induces pancreatic cancer cell apoptosis**. *Biochem Biophys Res Commun* 2001, **287**(2):522-529.

128. Elnemr A, Ohta T, Iwata K, Ninomia I, Fushida S, Nishimura G, Kitagawa H, Kayahara M, Yamamoto M, Terada T *et al*: **PPARgamma ligand (thiazolidinedione) induces growth arrest and differentiation markers of human pancreatic cancer cells**. *Int J Oncol* 2000, **17**(6):1157-1164.

129. Koga H, Sakisaka S, Harada M, Takagi T, Hanada S, Taniguchi E, Kawaguchi T, Sasatomi K, Kimura R, Hashimoto O *et al*: **Involvement of p21(WAF1/Cip1),**

p27(Kip1), and p18(INK4c) in troglitazone-induced cell-cycle arrest in human hepatoma cell lines. *Hepatology* 2001, **33**(5):1087-1097.

130. Itami A, Watanabe G, Shimada Y, Hashimoto Y, Kawamura J, Kato M, Hosotani R, Imamura M: **Ligands for peroxisome proliferator-activated receptor gamma inhibit growth of pancreatic cancers both in vitro and in vivo**. *Int J Cancer* 2001, **94**(3):370-376.

131. Panigrahy D, Singer S, Shen LQ, Butterfield CE, Freedman DA, Chen EJ, Moses MA, Kilroy S, Duensing S, Fletcher C *et al*: **PPARgamma ligands inhibit primary tumor growth and metastasis by inhibiting angiogenesis**. *J Clin Invest* 2002, **110**(7):923-932.

132. Jozkowicz A, Dulak J, Piatkowska E, Placha W, Dembinska-Kiec A: **Ligands of peroxisome proliferator-activated receptor-gamma increase the generation of vascular endothelial growth factor in vascular smooth muscle cells and in macrophages**. *Acta Biochim Pol* 2000, **47**(4):1147-1157.

133. Ishay-Ronen D, Diepenbruck M, Kalathur RKR, Sugiyama N, Tiede S, Ivanek R, Bantug G, Morini MF, Wang J, Hess C *et al*: **Gain Fat-Lose Metastasis: Converting Invasive Breast Cancer Cells into Adipocytes Inhibits Cancer Metastasis**. *Cancer Cell* 2019, **35**(1):17-32 e16.

134. Zaytseva YY, Wallis NK, Southard RC, Kilgore MW: **The PPARgamma antagonist T0070907 suppresses breast cancer cell proliferation and motility via both PPARgamma-dependent and -independent mechanisms**. *Anticancer Res* 2011, **31**(3):813-823.

135. Cheng S, Qian K, Wang Y, Wang G, Liu X, Xiao Y, Wang X: **PPARgamma inhibition regulates the cell cycle, proliferation and motility of bladder cancer cells**. *J Cell Mol Med* 2019, **23**(5):3724-3736.

136. Kitamura S, Miyazaki Y, Hiraoka S, Toyota M, Nagasawa Y, Kondo S, Kiyohara T, Shinomura Y, Matsuzawa Y: **PPARgamma inhibits the expression of c-MET in human gastric cancer cells through the suppression of Ets**. *Biochem Biophys Res Commun* 1999, **265**(2):453-456.

137. Debrock G, Vanhentenrijk V, Sciot R, Debiec-Rychter M, Oyen R, Van Oosterom A: **A phase II trial with rosiglitazone in liposarcoma patients**. *Br J Cancer* 2003, **89**(8):1409-1412.

138. Binienda A, Ziolkowska S, Pluciennik E: **The Anticancer Properties of Silibinin: Its Molecular Mechanism and Therapeutic Effect in Breast Cancer**. *Anticancer Agents Med Chem* 2020, **20**(15):1787-1796.

139. Chu SC, Chiou HL, Chen PN, Yang SF, Hsieh YS: **Silibinin inhibits the invasion of human lung cancer cells via decreased productions of urokinase-plasminogen activator and matrix metalloproteinase-2**. *Mol Carcinog* 2004, **40**(3):143-149.

140. Murali Iyangar R, Devaraj E: **Silibinin Triggers the Mitochondrial Pathway of Apoptosis in Human Oral Squamous Carcinoma Cells**. *Asian Pac J Cancer Prev* 2020, **21**(7):1877-1882.

141. Flaig TW, Glode M, Gustafson D, van Bokhoven A, Tao Y, Wilson S, Su LJ, Li Y, Harrison G, Agarwal R *et al*: **A study of high-dose oral silybin-phytosome followed by prostatectomy in patients with localized prostate cancer**. *Prostate* 2010, **70**(8):848-855.

142. Nambiar DK, Deep G, Singh RP, Agarwal C, Agarwal R: **Silibinin inhibits aberrant lipid metabolism, proliferation and emergence of androgen-independence in prostate cancer cells via primarily targeting the sterol response element binding protein 1**. *Oncotarget* 2014, **5**(20):10017-10033.

143. Brovkovych V, Izhar Y, Danes JM, Dubrovskyi O, Sakallioglu IT, Morrow LM, Atilla-Gokcumen GE, Frasor J: **Fatostatin induces pro- and anti-apoptotic lipid accumulation in breast cancer**. *Oncogenesis* 2018, **7**(8):66.

144. Li X, Chen YT, Hu P, Huang WC: **Fatostatin displays high antitumor activity in prostate cancer by blocking SREBP-regulated metabolic pathways and androgen receptor signaling**. *Mol Cancer Ther* 2014, **13**(4):855-866.

145. Kamisuki S, Shirakawa T, Kugimiya A, Abu-Elheiga L, Choo HY, Yamada K, Shimogawa H, Wakil SJ, Uesugi M: **Synthesis and evaluation of diarylthiazole derivatives that inhibit activation of sterol regulatory element-binding proteins**. *J Med Chem* 2011, **54**(13):4923-4927.

146. Guan M, Fousek K, Jiang C, Guo S, Synold T, Xi B, Shih CC, Chow WA: **Nelfinavir induces liposarcoma apoptosis through inhibition of regulated intramembrane proteolysis of SREBP-1 and ATF6**. *Clin Cancer Res* 2011, **17**(7):1796-1806.

147. Guan M, Su L, Yuan YC, Li H, Chow WA: **Nelfinavir and nelfinavir analogs block site-2 protease cleavage to inhibit castration-resistant prostate cancer**. *Sci Rep* 2015, **5**:9698.

148. Dean EJ, Falchook GS, Patel MR, Brenner AJ, Infante JR, Arkenau HT, Borazanci EH, Lopez JS, Pant S, Schmid P *et al*: **Preliminary activity in the first in human study of the first-in-class fatty acid synthase (FASN) inhibitor, TVB-2640.** *Journal of Clinical Oncology* 2016, **34**(15).

149. Menendez JA, Mehmi I, Atlas E, Colomer R, Lupu R: **Novel signaling molecules implicated in tumor-associated fatty acid synthase-dependent breast cancer cell proliferation and survival: Role of exogenous dietary fatty acids, p53-p21WAF1/CIP1, ERK1/2 MAPK, p27KIP1, BRCA1, and NF-kappaB**. *Int J Oncol* 2004, **24**(3):591-608.

150. Zhou W, Simpson PJ, McFadden JM, Townsend CA, Medghalchi SM, Vadlamudi A, Pinn ML, Ronnett GV, Kuhajda FP: **Fatty acid synthase inhibition triggers apoptosis during S phase in human cancer cells**. *Cancer Res* 2003, **63**(21):7330-7337.

151. Shiragami R, Murata S, Kosugi C, Tezuka T, Yamazaki M, Hirano A, Yoshimura Y, Suzuki M, Shuto K, Koda K: **Enhanced antitumor activity of cerulenin combined with oxaliplatin in human colon cancer cells**. *Int J Oncol* 2013, **43**(2):431-438.

152. Pizer ES, Wood FD, Heine HS, Romantsev FE, Pasternack GR, Kuhajda FP: **Inhibition of fatty acid synthesis delays disease progression in a xenograft model of ovarian cancer**. *Cancer Res* 1996, **56**(6):1189-1193.

153. Menendez JA, Oza BP, Atlas E, Verma VA, Mehmi I, Lupu R: **Inhibition of tumor-associated fatty acid synthase activity antagonizes estradiol- and tamoxifen-induced agonist transactivation of estrogen receptor (ER) in human endometrial adenocarcinoma cells**. *Oncogene* 2004, **23**(28):4945-4958.

154. Little JL, Wheeler FB, Fels DR, Koumenis C, Kridel SJ: **Inhibition of fatty acid synthase induces endoplasmic reticulum stress in tumor cells**. *Cancer Res* 2007, **67**(3):1262-1269.

155. Menendez JA, Vellon L, Lupu R: **Antitumoral actions of the anti-obesity drug orlistat (XenicalTM) in breast cancer cells: blockade of cell cycle progression, promotion of apoptotic cell death and PEA3-mediated transcriptional repression of Her2/neu (erbB-2) oncogene**. *Ann Oncol* 2005, **16**(8):1253-1267.

156. Beckers A, Organe S, Timmermans L, Scheys K, Peeters A, Brusselmans K, Verhoeven G, Swinnen JV: **Chemical inhibition of acetyl-CoA carboxylase induces growth arrest and cytotoxicity selectively in cancer cells**. *Cancer Res* 2007, **67**(17):8180-8187.

157. Svensson RU, Parker SJ, Eichner LJ, Kolar MJ, Wallace M, Brun SN, Lombardo PS, Van Nostrand JL, Hutchins A, Vera L *et al*: **Inhibition of acetyl-CoA carboxylase suppresses fatty acid synthesis and tumor growth of non-small-cell lung cancer in preclinical models**. *Nat Med* 2016, **22**(10):1108-1119.

158. Wang C, Xu C, Sun M, Luo D, Liao DF, Cao D: **Acetyl-CoA carboxylase-alpha inhibitor TOFA induces human cancer cell apoptosis**. *Biochem Biophys Res Commun* 2009, **385**(3):302-306.

159. Guseva NV, Rokhlin OW, Glover RA, Cohen MB: **TOFA (5-tetradecyl-oxy-2-furoic acid) reduces fatty acid synthesis, inhibits expression of AR, neuropilin-1 and Mcl-1 and kills prostate cancer cells independent of p53 status**. *Cancer Biol Ther* 2011, **12**(1):80-85.

160. Li S, Qiu L, Wu B, Shen H, Zhu J, Zhou L, Gu L, Di W: **TOFA suppresses ovarian cancer cell growth in vitro and in vivo**. *Mol Med Rep* 2013, **8**(2):373-378.

161. von Roemeling CA, Marlow LA, Wei JJ, Cooper SJ, Caulfield TR, Wu K, Tan WW, Tun HW, Copland JA: **Stearoyl-CoA Desaturase 1 Is a Novel Molecular Therapeutic Target for Clear Cell Renal Cell Carcinoma**. *Clinical Cancer Research* 2013, **19**(9):2368-2380.

162. She KL, Fang SH, Du W, Fan XX, He JX, Pan H, Huang LY, He P, Huang J: **SCD1 is required for EGFR-targeting cancer therapy of lung cancer via re-activation of EGFR/PI3K/AKT signals**. *Cancer Cell Int* 2019, **19**.

163. Chen L, Ren J, Yang LH, Li YT, Fu J, Li YH, Tian YF, Qiu FN, Liu ZG, Qiu Y: **Stearoyl-CoA desaturase-1 mediated cell apoptosis in colorectal cancer by promoting ceramide synthesis**. *Sci Rep-Uk* 2016, **6**.

164. Huang GM, Jiang QH, Cai C, Qu M, Shen W: **SCD1 negatively regulates autophagy-induced cell death in human hepatocellular carcinoma through inactivation of the AMPK signaling pathway**. *Cancer Letters* 2015, **358**(2):180-190.

165. Pinkham K, Park DJ, Hashemiaghdam A, Kirov AB, Adam I, Rosiak K, da Hora CC, Teng J, Cheah PS, Carvalho L *et al*: **Stearoyl CoA Desaturase Is Essential for Regulation of Endoplasmic Reticulum Homeostasis and Tumor Growth in Glioblastoma Cancer Stem Cells**. *Stem Cell Rep* 2019, **12**(4):712-727.

166. Li JJ, Condello S, Thomes-Pepin J, Ma XX, Xia Y, Hurley TD, Matei D, Cheng JX: **Lipid Desaturation Is a Metabolic Marker and Therapeutic Target of Ovarian Cancer Stem Cells**. *Cell Stem Cell* 2017, **20**(3):303-+.

167. Liu GH, Feng S, Jia L, Wang CY, Fu Y, Luo YZ: **Lung fibroblasts promote metastatic colonization through upregulation of stearoyl-CoA desaturase 1 in tumor cells**. *Oncogene* 2018, **37**(11):1519-1533.

168. Apostolova SN, Toshkova RA, Momchilova AB, Tzoneva RD: **Statins and Alkylphospholipids as New Anticancer Agents Targeting Lipid Metabolism**. *Anticancer Agents Med Chem* 2016, **16**(12):1512-1522.

169. Gong J, Sachdev E, Robbins LA, Lin E, Hendifar AE, Mita MM: **Statins and pancreatic cancer**. *Oncol Lett* 2017, **13**(3):1035-1040.

170. Cauley JA, McTiernan A, Rodabough RJ, LaCroix A, Bauer DC, Margolis KL, Paskett ED, Vitolins MZ, Furberg CD, Chlebowski RT *et al*: **Statin use and breast cancer: prospective results from the Women's Health Initiative**. *J Natl Cancer Inst* 2006, **98**(10):700-707.

171. El-Serag HB, Johnson ML, Hachem C, Morgana RO: **Statins are associated with a reduced risk of hepatocellular carcinoma in a large cohort of patients with diabetes**. *Gastroenterology* 2009, **136**(5):1601-1608.

172. Ahern TP, Pedersen L, Tarp M, Cronin-Fenton DP, Garne JP, Silliman RA, Sorensen HT, Lash TL: **Statin prescriptions and breast cancer recurrence risk: a Danish nationwide prospective cohort study**. *J Natl Cancer Inst* 2011, **103**(19):1461-1468.

173. Graaf MR, Beiderbeck AB, Egberts AC, Richel DJ, Guchelaar HJ: **The risk of cancer in users of statins**. *J Clin Oncol* 2004, **22**(12):2388-2394.

174. Zhuang L, Kim J, Adam RM, Solomon KR, Freeman MR: **Cholesterol targeting alters lipid raft composition and cell survival in prostate cancer cells and xenografts**. *J Clin Invest* 2005, **115**(4):959-968.

175. Emilsson L, Garcia-Albeniz X, Logan RW, Caniglia EC, Kalager M, Hernan MA: **Examining Bias in Studies of Statin Treatment and Survival in Patients With Cancer**. *JAMA Oncol* 2018, **4**(1):63-70.

176. Farooqi MAM, Malhotra N, Mukherjee SD, Sanger S, Dhesy-Thind SK, Ellis P, Leong DP: **Statin therapy in the treatment of active cancer: A systematic review and meta-analysis of randomized controlled trials**. *PLoS One* 2018, **13**(12):e0209486.

177. Nabors LB, Fiveash JB, Markert JM, Kekan MS, Gillespie GY, Huang Z, Johnson MJ, Meleth S, Kuo H, Gladson CL *et al*: **A phase 1 trial of ABT-510 concurrent with standard chemoradiation for patients with newly diagnosed glioblastoma**. *Arch Neurol* 2010, **67**(3):313-319.

178. Markovic SN, Suman VJ, Rao RA, Ingle JN, Kaur JS, Erickson LA, Pitot HC, Croghan GA, McWilliams RR, Merchan J *et al*: **A phase II study of ABT-510 (thrombospondin-1 analog) for the treatment of metastatic melanoma**. *Am J Clin Oncol* 2007, **30**(3):303-309.

179. Ebbinghaus S, Hussain M, Tannir N, Gordon M, Desai AA, Knight RA, Humerickhouse RA, Qian J, Gordon GB, Figlin R: **Phase 2 study of ABT-510 in patients with previously untreated advanced renal cell carcinoma**. *Clin Cancer Res* 2007, **13**(22 Pt 1):6689-6695.

180. Dawson DW, Volpert OV, Pearce SF, Schneider AJ, Silverstein RL, Henkin J, Bouck NP: **Three distinct D-amino acid substitutions confer potent antiangiogenic activity on an inactive peptide derived from a thrombospondin-1 type 1 repeat**. *Mol Pharmacol* 1999, **55**(2):332-338.

181. Isenberg JS, Yu C, Roberts DD: **Differential effects of ABT-510 and a CD36-binding peptide derived from the type 1 repeats of thrombospondin-1 on fatty acid uptake, nitric oxide signaling, and caspase activation in vascular cells**. *Biochem Pharmacol* 2008, **75**(4):875-882.

182. Al-Jameel W, Gou X, Jin X, Zhang J, Wei Q, Ai J, Li H, Al-Bayati A, Platt-Higgins A, Pettitt A *et al*: **Inactivated FABP5 suppresses malignant progression of prostate**

cancer cells by inhibiting the activation of nuclear fatty acid receptor PPARgamma. *Genes Cancer* 2019, **10**(3-4):80-96.

183. Carbonetti G, Converso C, Clement T, Wang C, Trotman LC, Ojima I, Kaczocha M: **Docetaxel/cabazitaxel and fatty acid binding protein 5 inhibitors produce synergistic inhibition of prostate cancer growth**. *Prostate* 2020, **80**(1):88-98.
184. Rao E, Singh P, Zhai X, Li Y, Zhu G, Zhang Y, Hao J, Chi YI, Brown RE, Cleary MP *et al*: **Inhibition of tumor growth by a newly-identified activator for epidermal fatty acid binding protein**. *Oncotarget* 2015, **6**(10):7815-7827.
185. Koltai T: **Nelfinavir and other protease inhibitors in cancer: mechanisms involved in anticancer activity**. *F1000Res* 2015, **4**:9.
186. Murata S, Yanagisawa K, Fukunaga K, Oda T, Kobayashi A, Sasaki R, Ohkohchi N: **Fatty acid synthase inhibitor cerulenin suppresses liver metastasis of colon cancer in mice**. *Cancer Sci* 2010, **101**(8):1861-1865.
187. Pischon T, Nothlings U, Boeing H: **Obesity and cancer**. *Proc Nutr Soc* 2008, **67**(2):128-145.
188. Larsson SC, Kumlin M, Ingelman-Sundberg M, Wolk A: **Dietary long-chain n-3 fatty acids for the prevention of cancer: a review of potential mechanisms**. *Am J Clin Nutr* 2004, **79**(6):935-945.
189. Ames BN, Gold LS, Willett WC: **The causes and prevention of cancer**. *Proc Natl Acad Sci U S A* 1995, **92**(12):5258-5265.
190. Huang LH, Chung HY, Su HM: **Docosahexaenoic acid reduces sterol regulatory element binding protein-1 and fatty acid synthase expression and inhibits cell proliferation by inhibiting pAkt signaling in a human breast cancer MCF-7 cell line**. *BMC Cancer* 2017, **17**(1):890.
191. Murad LB, da Silva Nogueira P, de Araujo WM, Sousa-Squiavinato ACM, Rocha MR, de Souza WF, de-Freitas-Junior J, Barcellos-de-Souza P, Bastos LG, Morgado-Diaz JA: **Docosahexaenoic acid promotes cell cycle arrest and decreases proliferation through WNT/beta-catenin modulation in colorectal cancer cells exposed to gamma-radiation**. *Biofactors* 2019, **45**(1):24-34.

192. Park M, Lim JW, Kim H: **Docoxahexaenoic Acid Induces Apoptosis of Pancreatic Cancer Cells by Suppressing Activation of STAT3 and NF-kappaB**. *Nutrients* 2018, **10**(11).

193. Serini S, Ottes Vasconcelos R, Fasano E, Calviello G: **Epigenetic regulation of gene expression and M2 macrophage polarization as new potential omega-3 polyunsaturated fatty acid targets in colon inflammation and cancer**. *Expert Opin Ther Targets* 2016, **20**(7):843-858.

194. Wang W, Yang J, Nimiya Y, Lee KSS, Sanidad K, Qi W, Sukamtoh E, Park Y, Liu Z, Zhang G: **omega-3 Polyunsaturated fatty acids and their cytochrome P450-derived metabolites suppress colorectal tumor development in mice**. *J Nutr Biochem* 2017, **48**:29-35.

195. Khadge S, Thiele GM, Sharp JG, McGuire TR, Klassen LW, Black PN, DiRusso CC, Cook L, Talmadge JE: **Long-chain omega-3 polyunsaturated fatty acids decrease mammary tumor growth, multiorgan metastasis and enhance survival**. *Clin Exp Metastasis* 2018, **35**(8):797-818.

196. Huang Q, Wen J, Chen G, Ge M, Gao Y, Ye X, Liu C, Cai C: **Omega-3 Polyunsaturated Fatty Acids Inhibited Tumor Growth via Preventing the Decrease of Genomic DNA Methylation in Colorectal Cancer Rats**. *Nutr Cancer* 2016, **68**(1):113-119.

197. Ding Y, Mullapudi B, Torres C, Mascarinas E, Mancinelli G, Diaz AM, McKinney R, Barron M, Schultz M, Heiferman M *et al*: **Omega-3 Fatty Acids Prevent Early Pancreatic Carcinogenesis via Repression of the AKT Pathway**. *Nutrients* 2018, **10**(9).

198. Notarnicola M, Tutino V, De Nunzio V, Dituri F, Caruso MG, Giannelli G: **Dietary omega-3 Polyunsaturated Fatty Acids Inhibit Tumor Growth in Transgenic Apc(Min/+) Mice, Correlating with CB1 Receptor Up-Regulation**. *Int J Mol Sci* 2017, **18**(3).

199. Zheng H, Tang H, Liu M, He M, Lai P, Dong H, Lin J, Jia C, Zhong M, Dai Y *et al*: **Inhibition of endometrial cancer by n-3 polyunsaturated fatty acids in preclinical models**. *Cancer Prev Res (Phila)* 2014, **7**(8):824-834.

200. Witte TR, Hardman WE: **The effects of omega-3 polyunsaturated Fatty Acid consumption on mammary carcinogenesis**. *Lipids* 2015, **50**(5):437-446.

201. Nindrea RD, Aryandono T, Lazuardi L, Dwiprahasto I: **Protective Effect of Omega-3 Fatty Acids in Fish Consumption Against Breast Cancer in Asian Patients: A Meta-Analysis**. *Asian Pac J Cancer Prev* 2019, **20**(2):327-332.

202. Schumacher MC, Laven B, Petersson F, Cederholm T, Onelov E, Ekman P, Brendler C: **A comparative study of tissue omega-6 and omega-3 polyunsaturated fatty acids (PUFA) in benign and malignant pathologic stage pT2a radical prostatectomy specimens**. *Urol Oncol* 2013, **31**(3):318-324.

203. Liu J, Li X, Hou J, Sun J, Guo N, Wang Z: **Dietary Intake of N-3 and N-6 Polyunsaturated Fatty Acids and Risk of Cancer: Meta-Analysis of Data from 32 Studies**. *Nutr Cancer* 2021, **73**(6):901-913.

204. Kim Y, Kim J: **N-6 Polyunsaturated Fatty Acids and Risk of Cancer: Accumulating Evidence from Prospective Studies**. *Nutrients* 2020, **12**(9).

205. Seo J, Jeong DW, Park JW, Lee KW, Fukuda J, Chun YS: **Fatty-acid-induced FABP5/HIF-1 reprograms lipid metabolism and enhances the proliferation of liver cancer cells**. *Commun Biol* 2020, **3**(1):638.

206. Xiang F, Wu K, Liu Y, Shi L, Wang D, Li G, Tao K, Wang G: **Omental adipocytes enhance the invasiveness of gastric cancer cells by oleic acid-induced activation of the PI3K-Akt signaling pathway**. *Int J Biochem Cell Biol* 2017, **84**:14-21.

207. Yang P, Su C, Luo X, Zeng H, Zhao L, Wei L, Zhang X, Varghese Z, Moorhead JF, Chen Y *et al*: **Dietary oleic acid-induced CD36 promotes cervical cancer cell growth and metastasis via up-regulation Src/ERK pathway**. *Cancer Lett* 2018, **438**:76-85.

208. Menendez JA, Vellon L, Colomer R, Lupu R: **Oleic acid, the main monounsaturated fatty acid of olive oil, suppresses Her-2/neu (erbB-2) expression and synergistically enhances the growth inhibitory effects of trastuzumab (Herceptin) in breast cancer cells with Her-2/neu oncogene amplification**. *Ann Oncol* 2005, **16**(3):359-371.

209. Jiang L, Wang W, He Q, Wu Y, Lu Z, Sun J, Liu Z, Shao Y, Wang A: **Oleic acid induces apoptosis and autophagy in the treatment of Tongue Squamous cell carcinomas**. *Sci Rep* 2017, **7**(1):11277.

210. Li S, Zhou T, Li C, Dai Z, Che D, Yao Y, Li L, Ma J, Yang X, Gao G: **High metastaticgastric and breast cancer cells consume oleic acid in an AMPK dependent manner**. *PLoS One* 2014, **9**(5):e97330.

211. Psaltopoulou T, Kosti RI, Haidopoulos D, Dimopoulos M, Panagiotakos DB: **Olive oil intake is inversely related to cancer prevalence: a systematic review and a meta-analysis of 13,800 patients and 23,340 controls in 19 observational studies**. *Lipids Health Dis* 2011, **10**:127.

212. Ruan L, Cheng SP, Zhu QX: **Dietary Fat Intake and the Risk of Skin Cancer: A Systematic Review and Meta-Analysis of Observational Studies**. *Nutr Cancer* 2020, **72**(3):398-408.

213. Sellem L, Srour B, Gueraud F, Pierre F, Kesse-Guyot E, Fiolet T, Lavalette C, Egnell M, Latino-Martel P, Fassier P *et al*: **Saturated, mono- and polyunsaturated fatty acid intake and cancer risk: results from the French prospective cohort NutriNet-Sante**. *Eur J Nutr* 2019, **58**(4):1515-1527.

214. Liss MA, Al-Bayati O, Gelfond J, Goros M, Ullevig S, DiGiovanni J, Hamilton-Reeves J, O'Keefe D, Bacich D, Weaver B *et al*: **Higher baseline dietary fat and fatty acid intake is associated with increased risk of incident prostate cancer in the SABOR study**. *Prostate Cancer Prostatic Dis* 2019, **22**(2):244-251.

215. Oppedisano F, Macri R, Gliozzi M, Musolino V, Carresi C, Maiuolo J, Bosco F, Nucera S, Caterina Zito M, Guarnieri L *et al*: **The Anti-Inflammatory and Antioxidant Properties of n-3 PUFAs: Their Role in Cardiovascular Protection**. *Biomedicines* 2020, **8**(9).

216. Liput KP, Lepczynski A, Ogluszka M, Nawrocka A, Polawska E, Grzesiak A, Slaska B, Pareek CS, Czarnik U, Pierzchala M: **Effects of Dietary n-3 and n-6 Polyunsaturated Fatty Acids in Inflammation and Cancerogenesis**. *Int J Mol Sci* 2021, **22**(13).

217. Tiwary S, Berzofsky JA, Terabe M: **Altered Lipid Tumor Environment and Its Potential Effects on NKT Cell Function in Tumor Immunity**. *Front Immunol* 2019, **10**:2187.

218. Miska J, Lee-Chang C, Rashidi A, Muroski ME, Chang AL, Lopez-Rosas A, Zhang P, Panek WK, Cordero A, Han Y *et al*: **HIF-1alpha Is a Metabolic Switch between**

Glycolytic-Driven Migration and Oxidative Phosphorylation-Driven Immunosuppression of Tregs in Glioblastoma. *Cell Rep* 2019, **27**(1):226-237 e224.

219. Zhang Y, Kurupati R, Liu L, Zhou XY, Zhang G, Hudaihed A, Filisio F, Giles-Davis W, Xu X, Karakousis GC *et al*: **Enhancing CD8(+) T Cell Fatty Acid Catabolism within a Metabolically Challenging Tumor Microenvironment Increases the Efficacy of Melanoma Immunotherapy**. *Cancer Cell* 2017, **32**(3):377-391 e379.
220. Mock A, Zschabitz S, Kirsten R, Scheffler M, Wolf B, Herold-Mende C, Kramer R, Busch E, Jenzer M, Jager D *et al*: **Serum very long-chain fatty acid-containing lipids predict response to immune checkpoint inhibitors in urological cancers**. *Cancer Immunol Immunother* 2019, **68**(12):2005-2014.
221. Smith PM, Howitt MR, Panikov N, Michaud M, Gallini CA, Bohlooly YM, Glickman JN, Garrett WS: **The microbial metabolites, short-chain fatty acids, regulate colonic Treg cell homeostasis**. *Science* 2013, **341**(6145):569-573.
222. Nomura M, Nagatomo R, Doi K, Shimizu J, Baba K, Saito T, Matsumoto S, Inoue K, Muto M: **Association of Short-Chain Fatty Acids in the Gut Microbiome With Clinical Response to Treatment With Nivolumab or Pembrolizumab in Patients With Solid Cancer Tumors**. *JAMA Netw Open* 2020, **3**(4):e202895.
223. Botticelli A, Vernocchi P, Marini F, Quagliariello A, Cerbelli B, Reddel S, Del Chierico F, Di Pietro F, Giusti R, Tomassini A *et al*: **Gut metabolomics profiling of non-small cell lung cancer (NSCLC) patients under immunotherapy treatment**. *J Transl Med* 2020, **18**(1):49.
224. Yang W, Bai Y, Xiong Y, Zhang J, Chen S, Zheng X, Meng X, Li L, Wang J, Xu C *et al*: **Potentiating the antitumour response of CD8(+) T cells by modulating cholesterol metabolism**. *Nature* 2016, **531**(7596):651-655.
225. Ma X, Bi E, Lu Y, Su P, Huang C, Liu L, Wang Q, Yang M, Kalady MF, Qian J *et al*: **Cholesterol Induces CD8(+) T Cell Exhaustion in the Tumor Microenvironment**. *Cell Metab* 2019, **30**(1):143-156 e145.
226. Beauchamp E, Yap MC, Iyer A, Perinpanayagam MA, Gamma JM, Vincent KM, Lakshmanan M, Raju A, Tergaonkar V, Tan SY *et al*: **Targeting N-myristoylation for therapy of B-cell lymphomas**. *Nat Commun* 2020, **11**(1):5348.

227. Nam GH, Kwon M, Jung H, Ko E, Kim SA, Choi Y, Song SJ, Kim S, Lee Y, Kim GB *et al*: **Statin-mediated inhibition of RAS prenylation activates ER stress to enhance the immunogenicity of KRAS mutant cancer**. *J Immunother Cancer* 2021, **9**(7).

228. Yao H, Lan J, Li C, Shi H, Brosseau JP, Wang H, Lu H, Fang C, Zhang Y, Liang L *et al*: **Inhibiting PD-L1 palmitoylation enhances T-cell immune responses against tumours**. *Nat Biomed Eng* 2019, **3**(4):306-317.

229. Yang Y, Hsu JM, Sun L, Chan LC, Li CW, Hsu JL, Wei Y, Xia W, Hou J, Qiu Y *et al*: **Palmitoylation stabilizes PD-L1 to promote breast tumor growth**. *Cell Res* 2019, **29**(1):83-86.

230. Sharma C, Wang HX, Li Q, Knoblich K, Reisenbichler ES, Richardson AL, Hemler ME: **Protein Acyltransferase DHHC3 Regulates Breast Tumor Growth, Oxidative Stress, and Senescence**. *Cancer Res* 2017, **77**(24):6880-6890.

231. Du W, Hua F, Li X, Zhang J, Li S, Wang W, Zhou J, Wang W, Liao P, Yan Y *et al*: **Loss of Optineurin Drives Cancer Immune Evasion via Palmitoylation-Dependent IFNGR1 Lysosomal Sorting and Degradation**. *Cancer Discov* 2021, **11**(7):1826-1843.

232. Robert C: **A decade of immune-checkpoint inhibitors in cancer therapy**. *Nat Commun* 2020, **11**(1):3801.

233. Wu HW, Volponi JV, Oliver AE, Parikh AN, Simmons BA, Singh S: **In vivo lipidomics using single-cell Raman spectroscopy**. *P Natl Acad Sci USA* 2011, **108**(9):3809-3814.

234. Tyurina YY, Tyurin VA, Anthonymuthu T, Amoscato AA, Sparvero LJ, Nesterova AM, Baynard ML, Sun W, He R, Khaitovich P *et al*: **"Redox lipidomics technology: Looking for a needle in a haystack"**. *Chem Phys Lipids* 2019, **221**:93-107.

235. Sun SC: **The non-canonical NF-kappaB pathway in immunity and inflammation**. *Nat Rev Immunol* 2017, **17**(9):545-558.

236. Yu H, Pardoll D, Jove R: **STATs in cancer inflammation and immunity: a leading role for STAT3**. *Nat Rev Cancer* 2009, **9**(11):798-809.

237. Qiao Y, He H, Jonsson P, Sinha I, Zhao C, Dahlman-Wright K: **AP-1 is a key regulator of proinflammatory cytokine TNFalpha-mediated triple-negative breast cancer progression**. *J Biol Chem* 2016, **291**(35):18309.

238. Semenza GL: **Targeting HIF-1 for cancer therapy**. *Nat Rev Cancer* 2003, **3**(10):721-732.

239. Zhang XH, Giuliano M, Trivedi MV, Schiff R, Osborne CK: **Metastasis dormancy in estrogen receptor-positive breast cancer**. *Clin Cancer Res* 2013, **19**(23):6389-6397.

240. Sanchez-Tillo E, de Barrios O, Siles L, Cuatrecasas M, Castells A, Postigo A: **beta-catenin/TCF4 complex induces the epithelial-to-mesenchymal transition (EMT)-activator ZEB1 to regulate tumor invasiveness**. *Proc Natl Acad Sci U S A* 2011, **108**(48):19204-19209.

241. Niehrs C, Acebron SP: **Mitotic and mitogenic Wnt signalling**. *EMBO J* 2012, **31**(12):2705-2713.

242. Hermeking H, Rago C, Schuhmacher M, Li Q, Barrett JF, Obaya AJ, O'Connell BC, Mateyak MK, Tam W, Kohlhuber F *et al*: **Identification of CDK4 as a target of c-MYC**. *Proc Natl Acad Sci U S A* 2000, **97**(5):2229-2234.

243. Leone G, Sears R, Huang E, Rempel R, Nuckolls F, Park CH, Giangrande P, Wu L, Saavedra HI, Field SJ *et al*: **Myc requires distinct E2F activities to induce S phase and apoptosis**. *Mol Cell* 2001, **8**(1):105-113.

244. Cam H, Dynlacht BD: **Emerging roles for E2F: beyond the G1/S transition and DNA replication**. *Cancer Cell* 2003, **3**(4):311-316.

245. Kent LN, Leone G: **The broken cycle: E2F dysfunction in cancer**. *Nat Rev Cancer* 2019, **19**(6):326-338.

246. Wang H, Wang X, Xu L, Zhang J, Cao H: **Integrated analysis of the E2F transcription factors across cancer types**. *Oncol Rep* 2020, **43**(4):1133-1146.

LHAD_manuscript

ORIGINALITY REPORT

11%
SIMILARITY INDEX

PRIMARY SOURCES

1	www.dovepress.com<br>Internet	102 words — 1%
2	"Regulation of Cancer Immune Checkpoints", Springer Science and Business Media LLC, 2020<br>Crossref	87 words — 1%
3	www.ncbi.nlm.nih.gov<br>Internet	68 words — 1%
4	repositorium.sdum.uminho.pt<br>Internet	55 words — 1%
5	www.nature.com<br>Internet	52 words — 1%
6	cwww.intechopen.com<br>Internet	50 words — 1%
7	Currie, Erin, Almut Schulze, Rudolf Zechner, Tobias C. Walther, and Robert V. Farese. "Cellular Fatty Acid Metabolism and Cancer", Cell Metabolism, 2013.<br>Crossref	46 words — 1%
8	atvb.ahajournals.org<br>Internet	43 words — 1%

9	Youngyo Kim, Jeongseon Kim. "N-6 Polyunsaturated Fatty Acids and Risk of Cancer: Accumulating Evidence from Prospective Studies", Nutrients, 2020<br>Crossref	34 words — < 1%
10	Hervé Guillou, Pascal G.P. Martin, Thierry Pineau. "Chapter 1 Transcriptional Regulation of Hepatic Fatty Acid Metabolism", Springer Science and Business Media LLC, 2008<br>Crossref	33 words — < 1%
11	onlinelibrary.wiley.com<br>Internet	33 words — < 1%
12	www.intechopen.com<br>Internet	32 words — < 1%
13	Turrado, Carlos, Teresa Puig, Javier García-Cárceles, Marta Artola, Bellinda Benhamú, Silvia Ortega-Gutiérrez, Joana Relat, Gloria Oliveras, Adriana Blancafort, Diego Haro, Pedro F. Marrero, Ramón Colomer, and María L. López-Rodríguez. "New Synthetic Inhibitors of Fatty Acid Synthase with Anticancer Activity", Journal of Medicinal Chemistry, 2012.<br>Crossref	27 words — < 1%
14	Carla Riera-Domingo, Annette Audigé, Sara Granja, Wan-Chen Cheng et al. "Immunity, Hypoxia and Metabolism - the ménage à trois of cancer: implications for immunotherapy", Physiological Reviews, 2019<br>Crossref	25 words — < 1%
15	Musso, G.. "Recent insights into hepatic lipid metabolism in non-alcoholic fatty liver disease (NAFLD)", Progress in Lipid Research, 200901<br>Crossref	24 words — < 1%

16 Obesity Inflammation and Cancer, 2013.
Crossref
24 words — < 1%

17 Xin Han, YanLing Wu, Qiao Yang, Gang Cao. "Peroxisome proliferator-activated receptors in the pathogenesis and therapies of liver fibrosis", Pharmacology & Therapeutics, 2020
Crossref
22 words — < 1%

18 eprints.gla.ac.uk
Internet
22 words — < 1%

19 Mitochondria The Anti- cancer Target for the Third Millennium, 2014.
Crossref
19 words — < 1%

20 Wenfeng Zeng, Xiaozhe Yin, Yunhan Jiang, Lingtao Jin, Wei Liang. "PPARα at the crossroad of metabolic–immune regulation in cancer", The FEBS Journal, 2021
Crossref
17 words — < 1%

21 Rosa, R.. "Biochemical changes during the embryonic development of Norway lobster, Nephrops norvegicus", Aquaculture, 20030501
Crossref
16 words — < 1%

22 Alfaia, António, Cristina M. Alfaia, Luís Patarata, Maria J. Fernandes, Maria H. Fernandes, Miguel Elias, Maria H. Ribeiro, and Maria J. Fraqueza. "Binomial effects of high isostatic pressure and time on the microbiological, sensory characteristics and lipid composition stability of vacuum packed dry fermented sausages “chouriço”", Innovative Food Science & Emerging Technologies, 2015.
Crossref
14 words — < 1%

23	Chloe Choi, David K. Finlay. "Diverse Immunoregulatory Roles of Oxysterols—The Oxidized Cholesterol Metabolites", Metabolites, 2020<br>Crossref	14 words — < 1%
24	tessera.spandidos-publications.com<br>Internet	12 words — < 1%
25	docplayer.net<br>Internet	11 words — < 1%
26	Na Li, Zhang-Sen Zhou, Yang Shen, Jie Xu, Hong-Hua Miao, Ying Xiong, Feng Xu, Bo-Liang Li, Jie Luo, Bao-Liang Song. "Inhibition of the sterol regulatory element-binding protein pathway suppresses hepatocellular carcinoma by repressing inflammation in mice", Hepatology, 2017<br>Crossref	9 words — < 1%
27	Zhao, J.. "Deposition of docosahexaenoic acid (DHA) is limited in forebrain of young obese fa/fa Zucker rats fed a diet high in @a-linolenic acid but devoid of DHA", The Journal of Nutritional Biochemistry, 201109<br>Crossref	9 words — < 1%
28	Fabiana A Rossi, Juliana H Enriqué Steinberg, Ezequiel H Calvo Roitberg, Molishree Joshi et al. "USP19 modulates cancer cell migration and invasion and acts as a novel prognostic marker in patients with early breast cancer", Cold Spring Harbor Laboratory, 2020<br>Crossref Posted Content	8 words — < 1%
29	Xiu Nie, Liujia Qian, Rui Sun, Bo Huang et al. "Multi-organ Proteomic Landscape of COVID-19 Autopsies", Cold Spring Harbor Laboratory, 2020<br>Crossref Posted Content	6 words — < 1%

EXCLUDE QUOTES	ON	EXCLUDE MATCHES	OFF
EXCLUDE BIBLIOGRAPHY	ON		
